# Supplementary material for: A cell-based evaluation of a non-essential amino acid formulation as a non-bioactive control for activation and stimulation of muscle protein synthesis using ex vivo human serum
Source: PLoS One. 2019 Nov 19;14(11):e0220757. doi: 10.1371/journal.pone.0220757 (PMC6863517; doi:10.1371/journal.pone.0220757)
Supplement: S1 File — Raw, uncropped images of all western blots used for quantification of Puromycin, Total protein, pmTOR, Total mTOR, pP70S6K, Total P70S6K, p4E-BP1, and Total 4E-BP1 with identified and labelled lanes for each condition. (PDF) [file pone.0220757.s001.pdf]

Title: A cell-based evaluation of a non-essential amino acid formulation as a non-bioactive control for activation and stimulation of muscle protein synthesis using *ex vivo* human serum

Authors: Bijal Patel[a,b,c], Martina Pauk[a,b,c], Miryam Amigo-Benavent[a,b,c,d], Alice B. Nongonierma[a,e], Richard J. Fitzgerald[a,e], Philip M. Jakeman[a,b,c,d], Brian P. Carson[a,b,c,d].

Original Western Blot Images

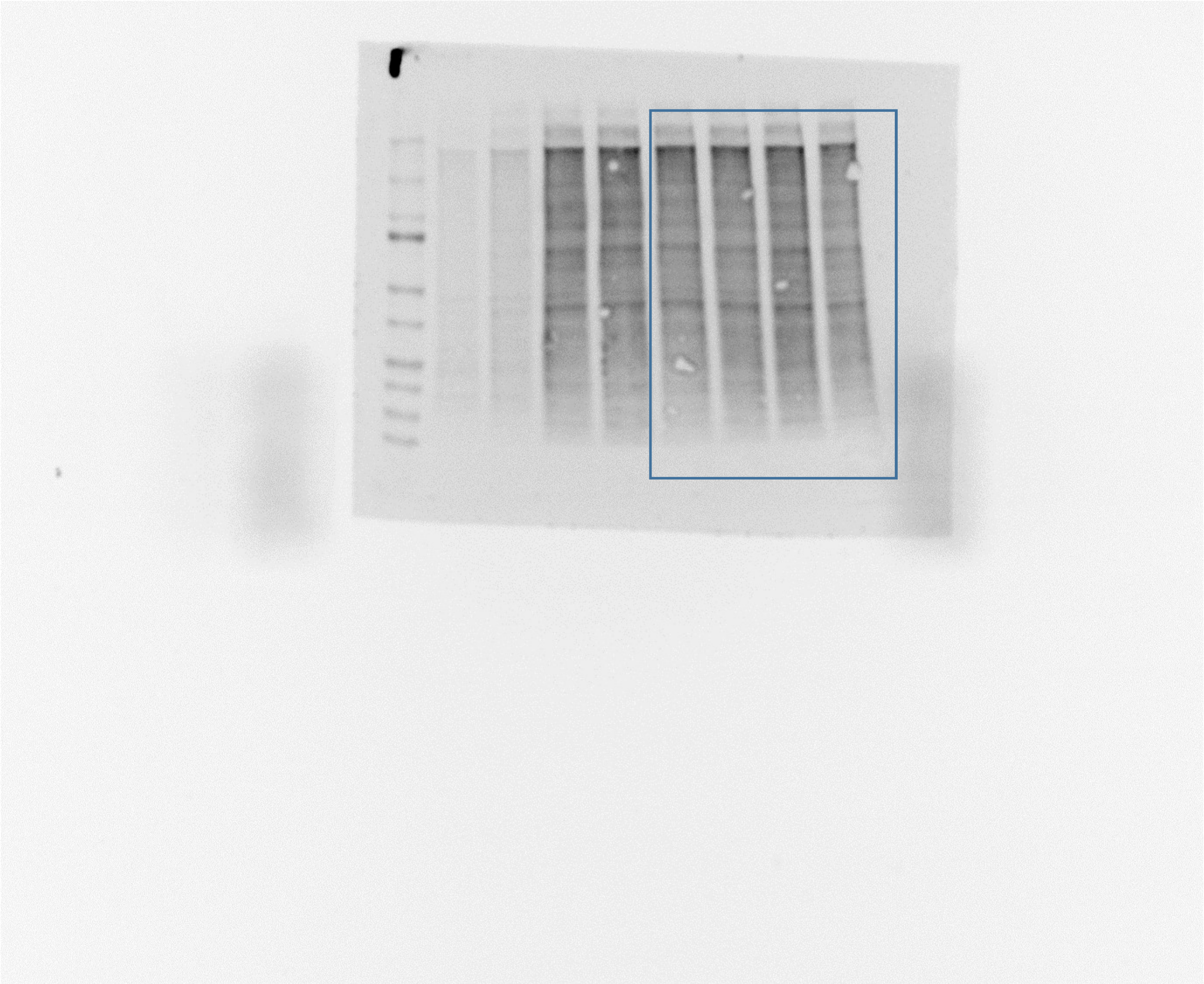

Puromycin Participant 1

Lane 5 – WPH FAST

Lane 6 – WPH Fed

Lane 7 – NEAA FAST

Lane 8 – NEAA Fed

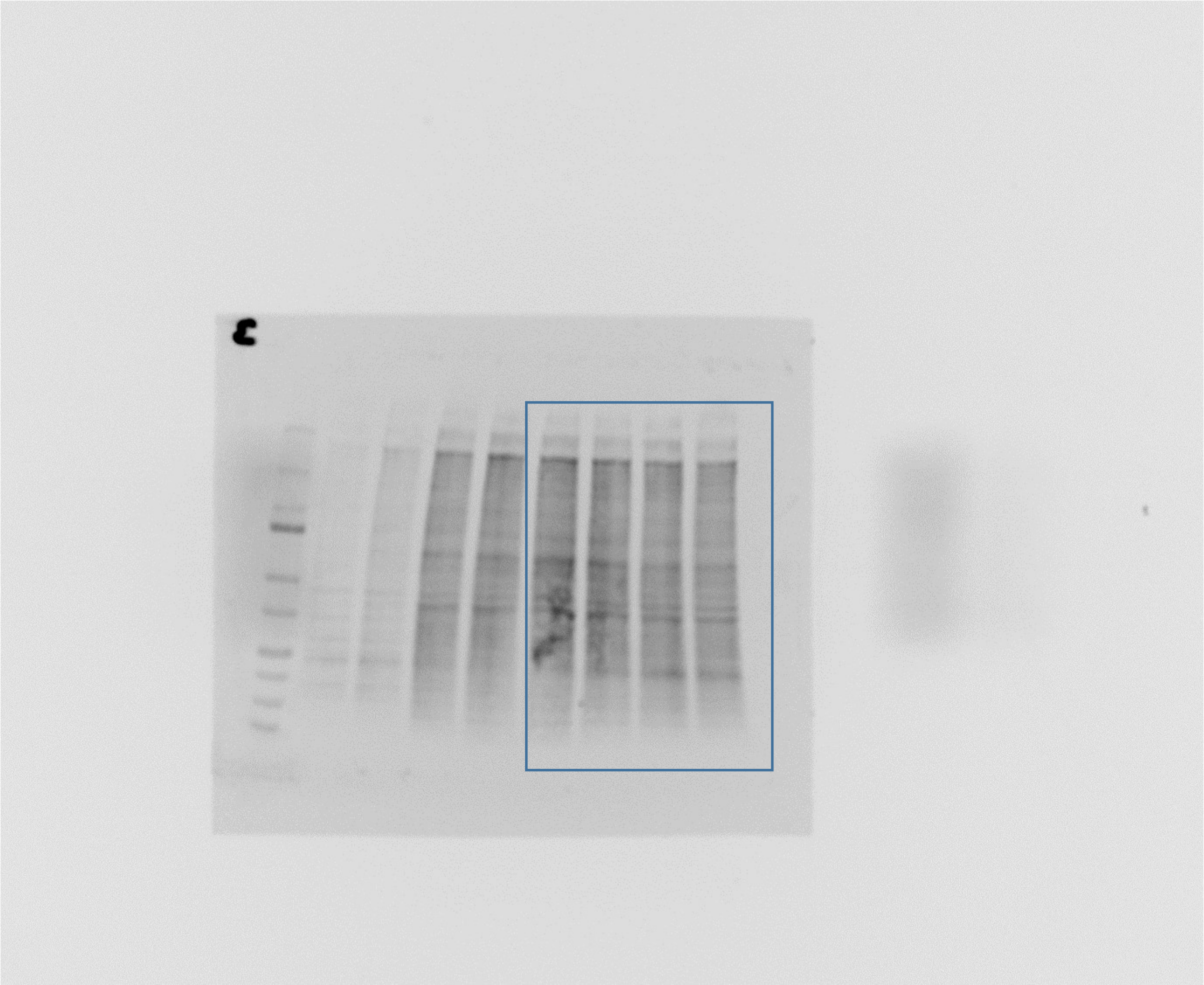

Puromycin Participant 2

Lane 5 – WPH FAST

Lane 6 – WPH Fed

Lane 7 – NEAA FAST

Lane 8 – NEAA Fed

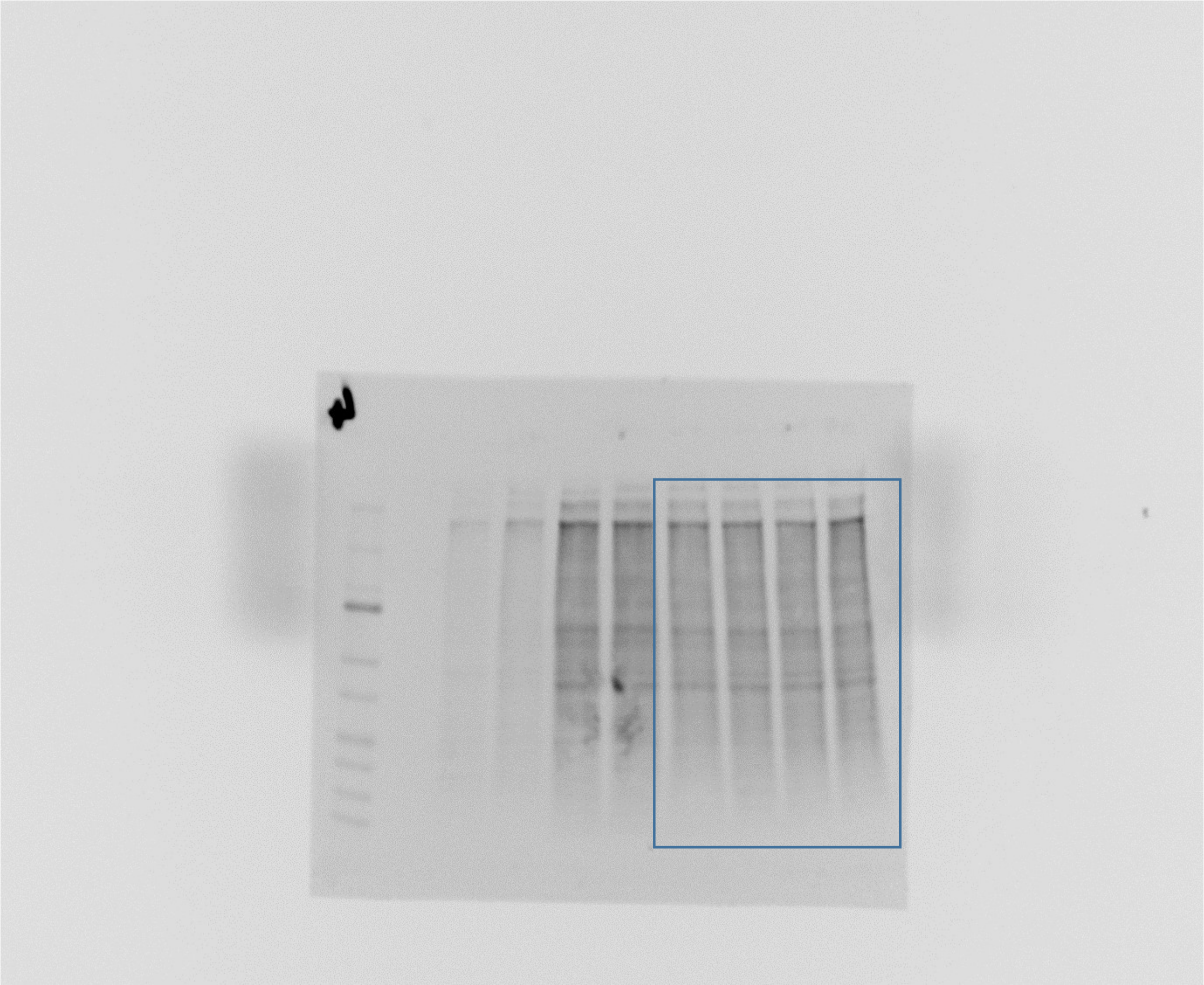

Puromycin Participant 3

Lane 5 – WPH FAST

Lane 6 – WPH Fed

Lane 7 – NEAA FAST

Lane 8 – NEAA Fed

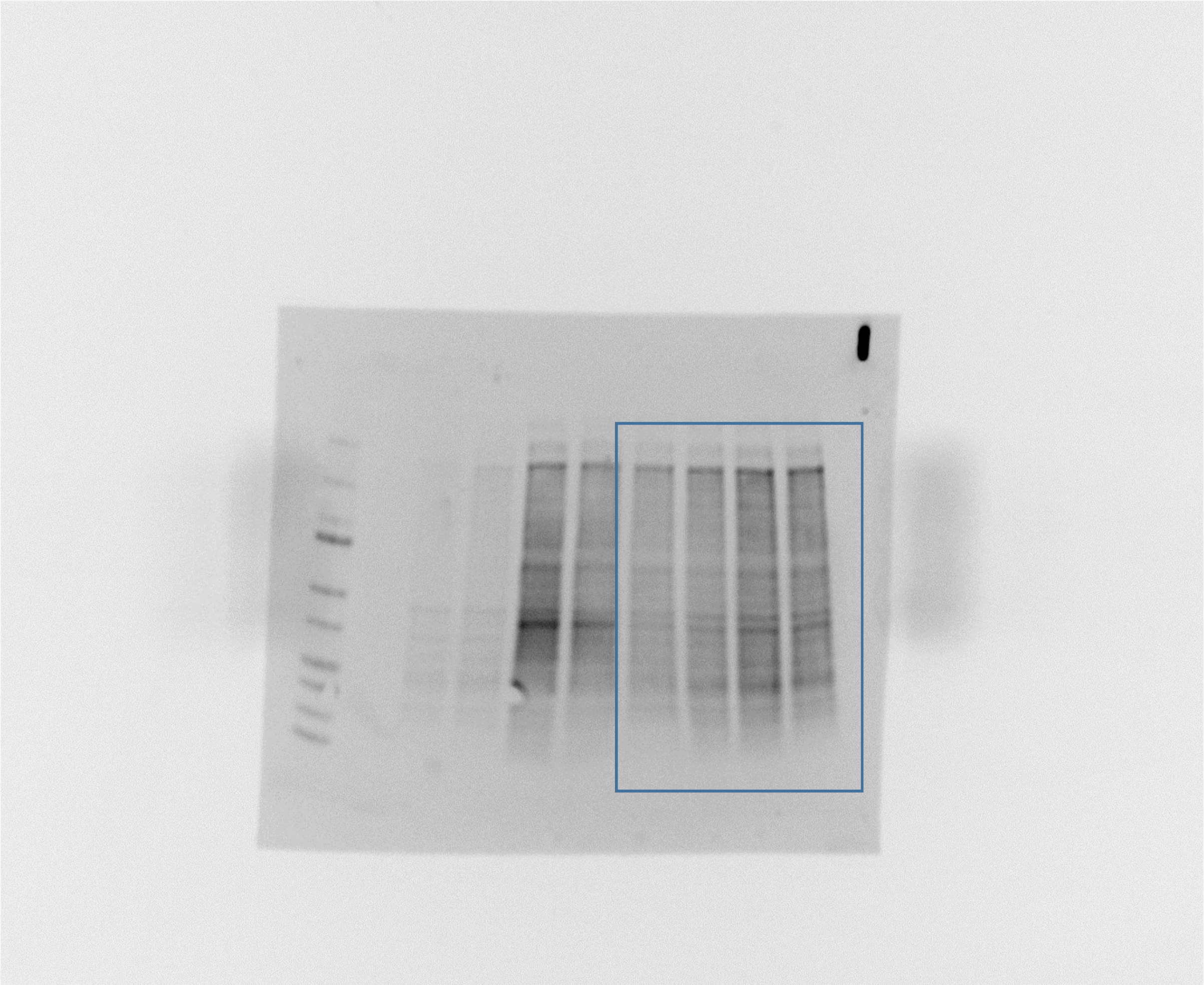

Puromycin Participant 4

Lane 5 – WPH FAST

Lane 6 – WPH Fed

Lane 7 – NEAA FAST

Lane 8 – NEAA Fed

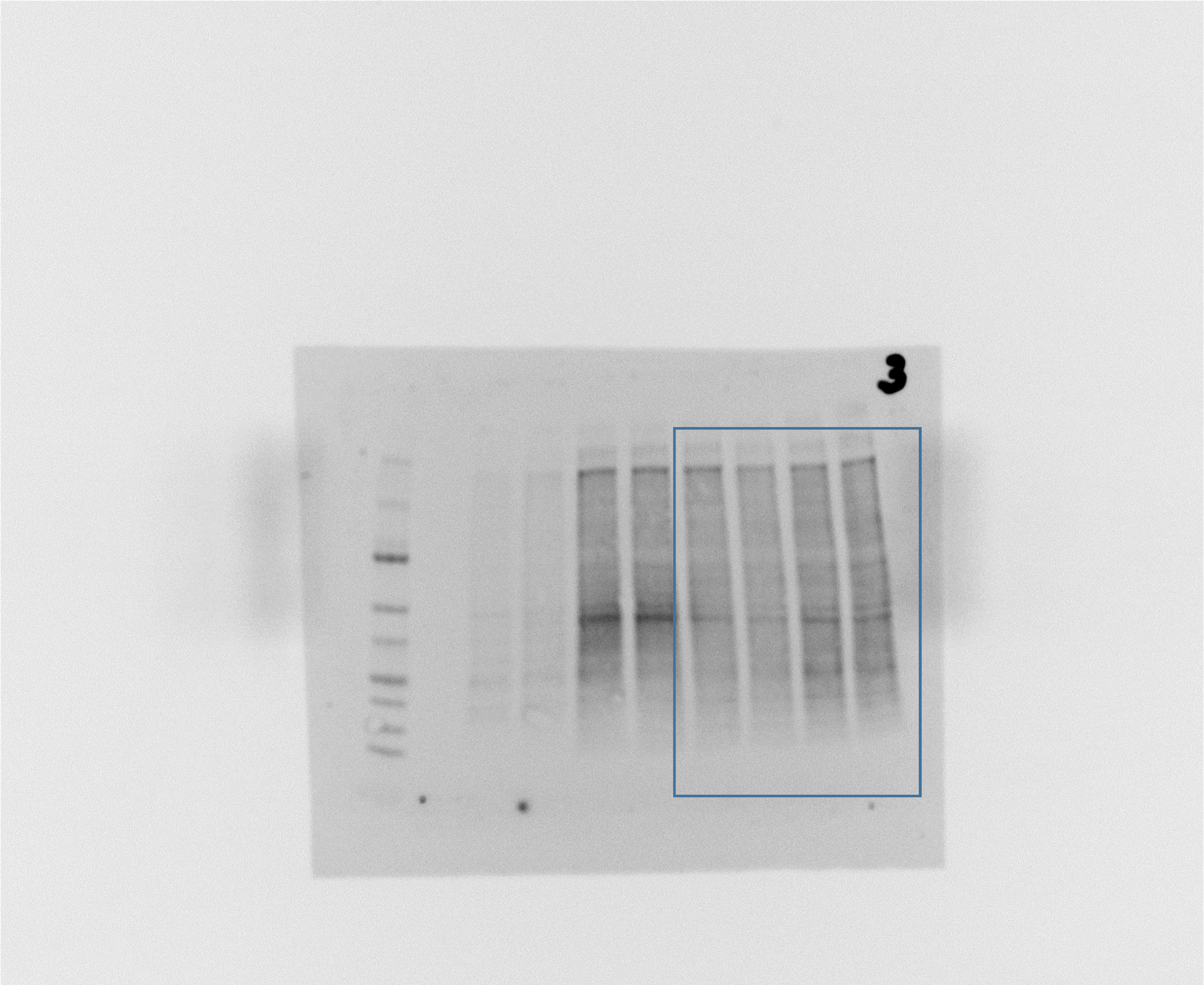

Puromycin Participant 5

Lane 5 – WPH FAST

Lane 6 – WPH Fed

Lane 7 – NEAA FAST

Lane 8 – NEAA Fed

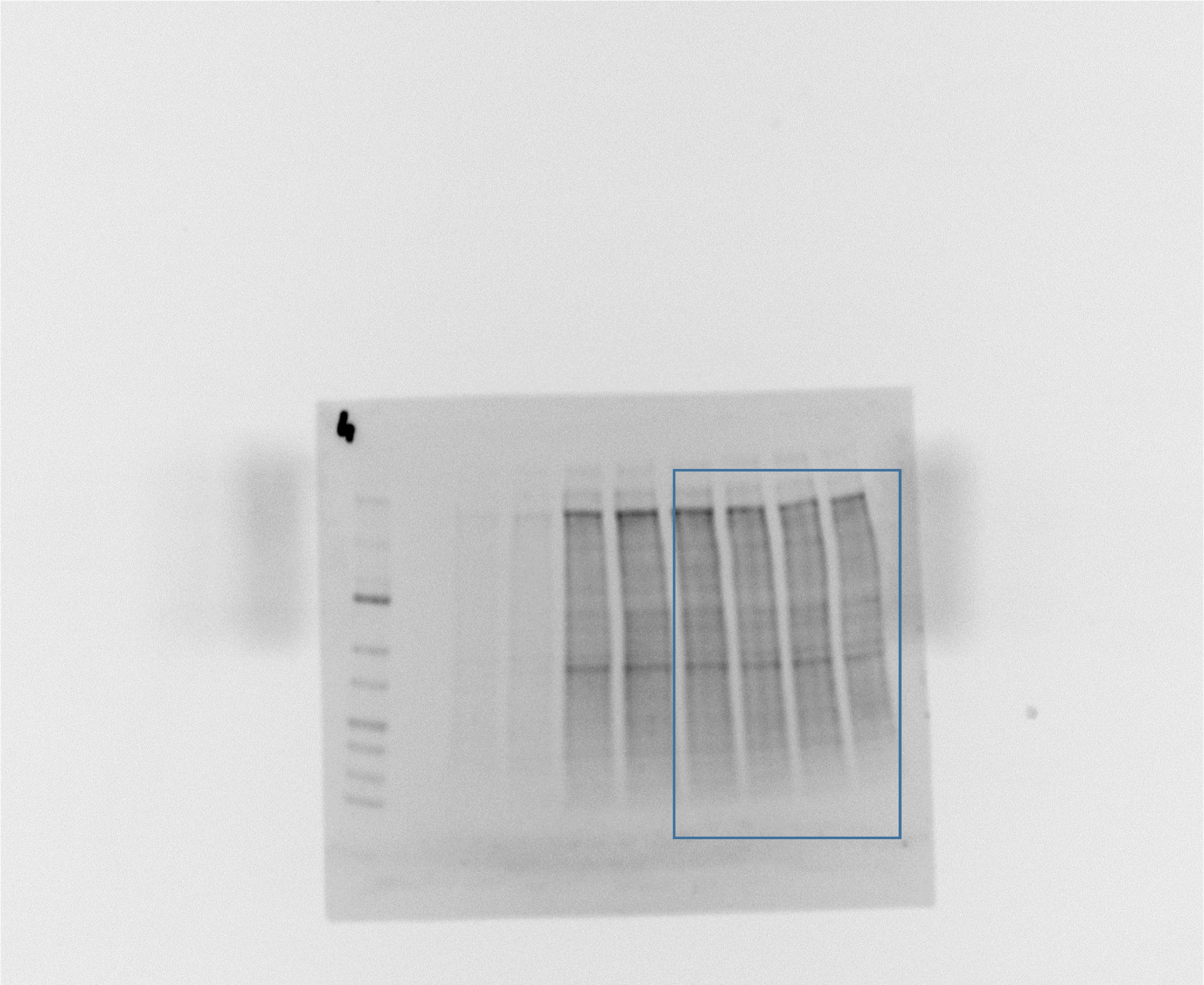

Puromycin Participant 6

Lane 5 – WPH FAST

Lane 6 – WPH Fed

Lane 7 – NEAA FAST

Lane 8 – NEAA Fed

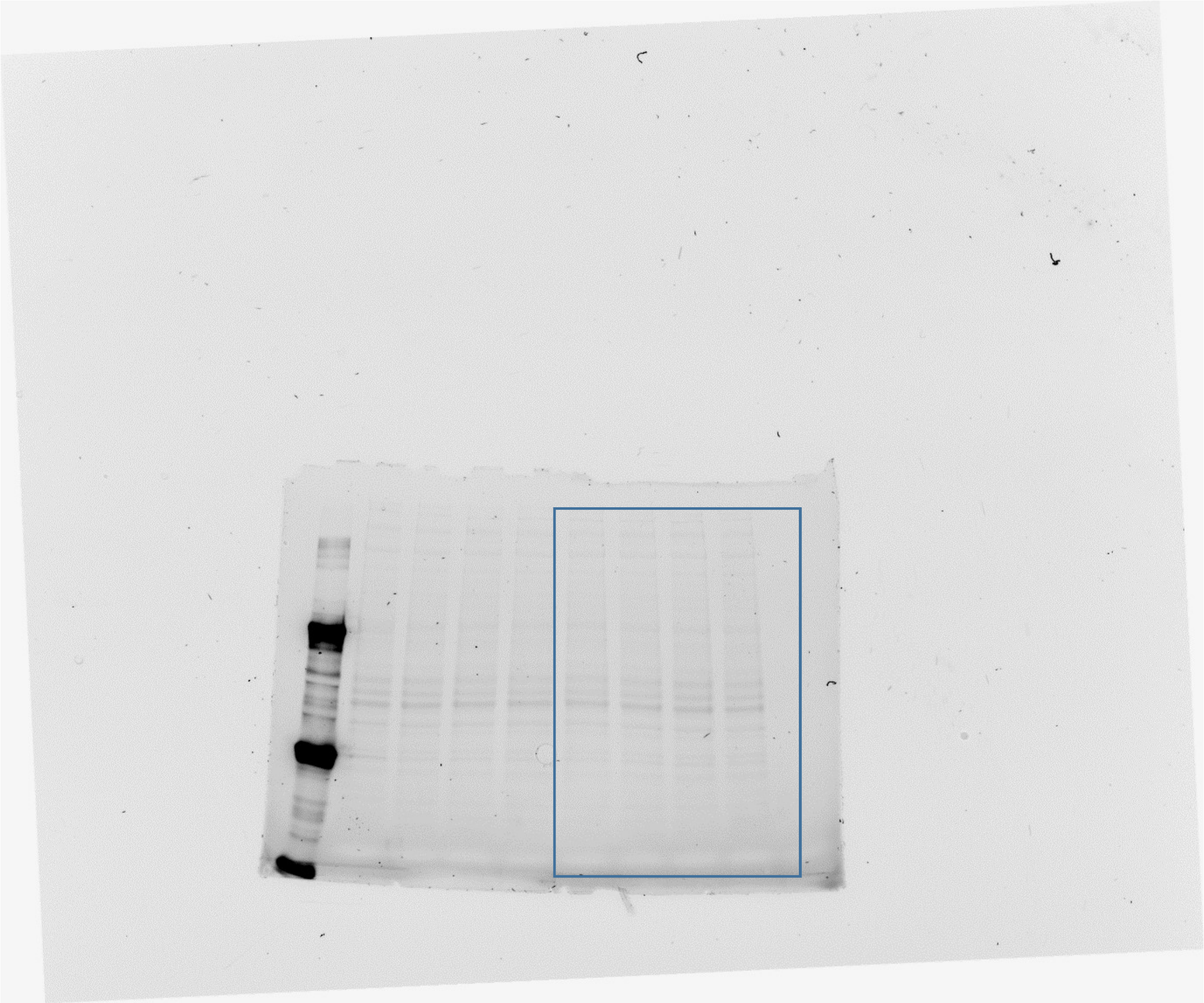

Total Protein Loading Control Participant 1 (Stain-free activated gel)

Lane 5 – WPH FAST

Lane 6 – WPH Fed

Lane 7 – NEAA FAST

Lane 8 – NEAA Fed

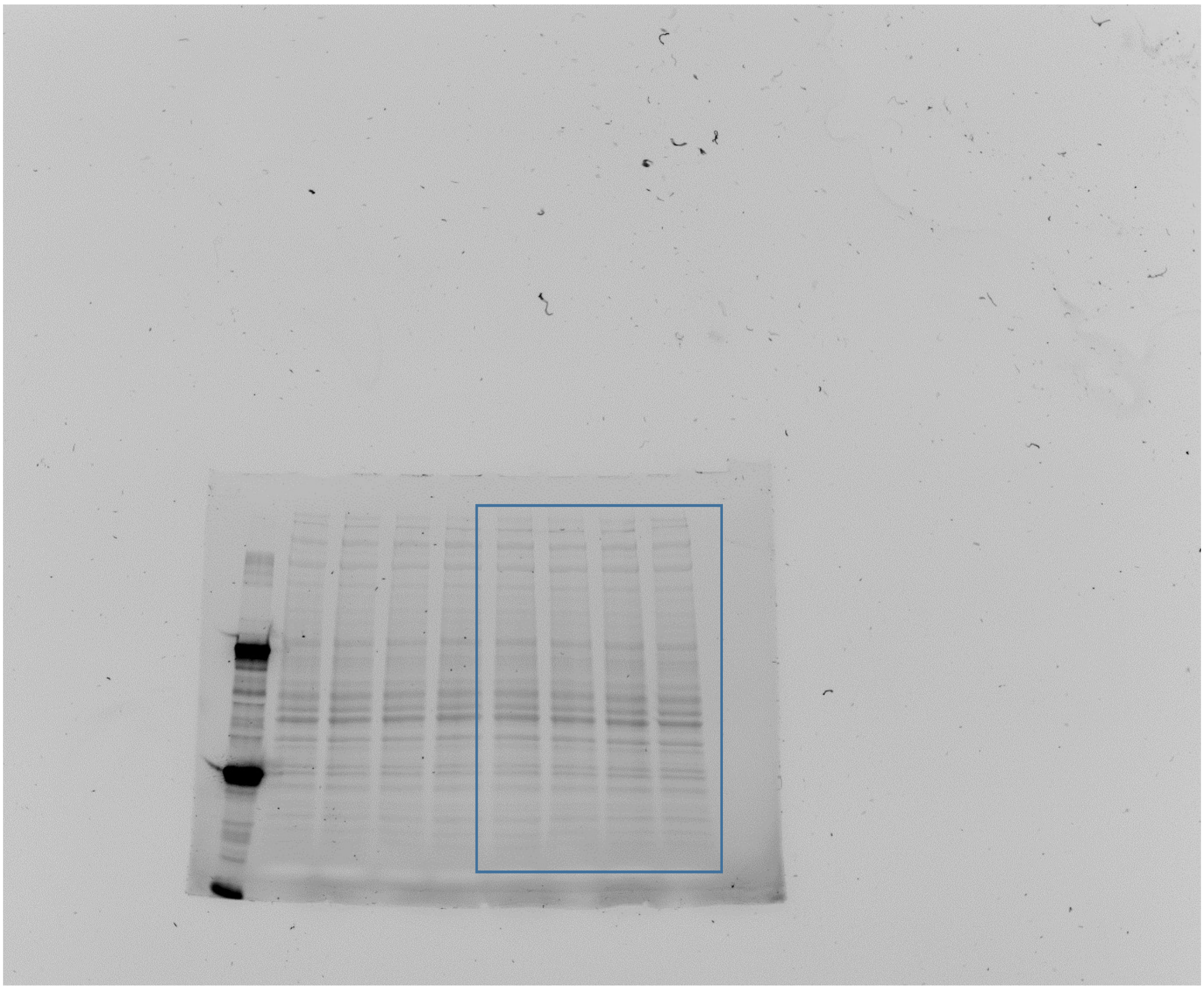

Total Protein Loading Control Participant 2 (Stain-free activated gel)

Lane 5 – WPH FAST

Lane 6 – WPH Fed

Lane 7 – NEAA FAST

Lane 8 – NEAA Fed

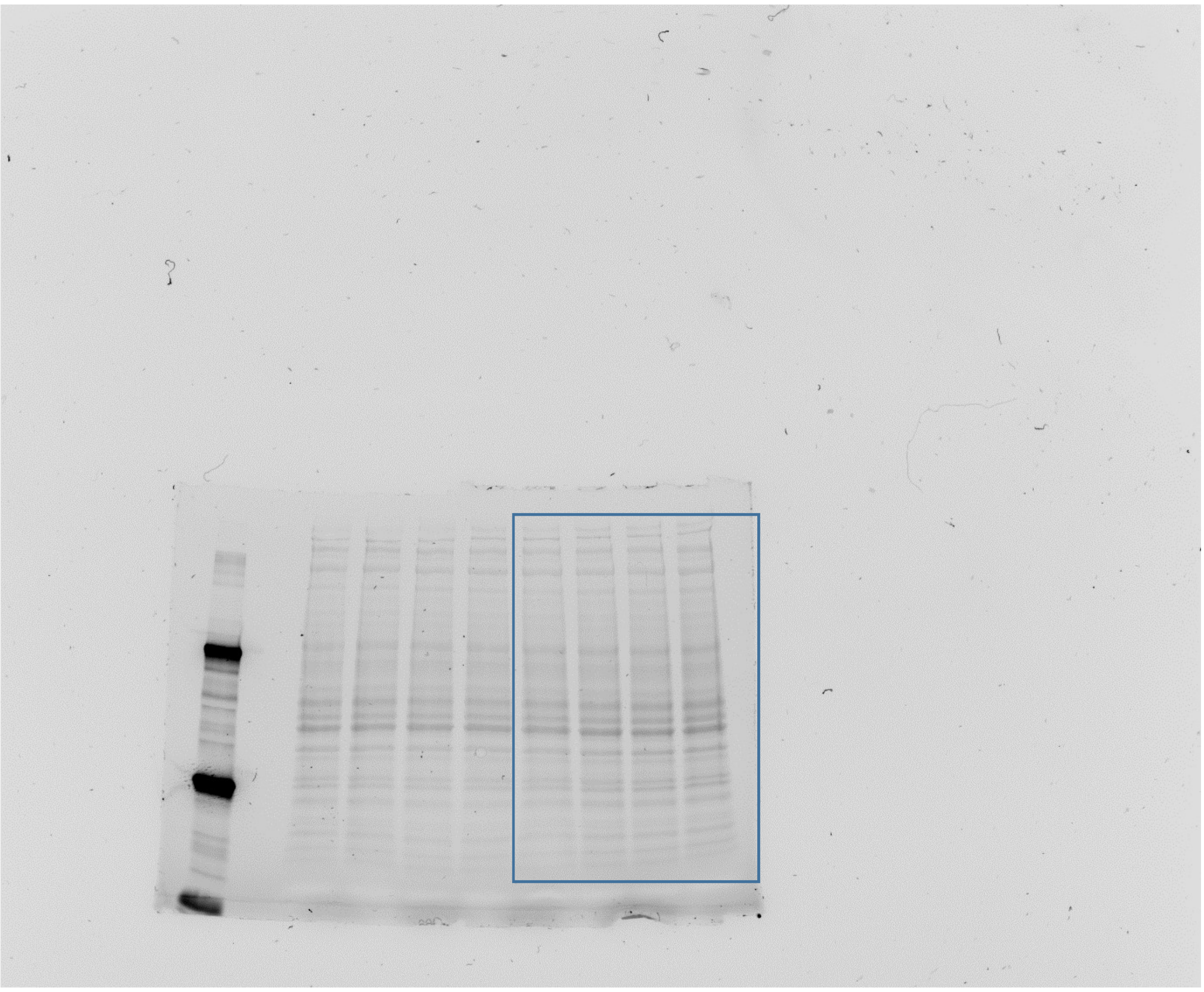

Total Protein Loading Control Participant 3 (Stain-free activated gel)

Lane 5 – WPH FAST

Lane 6 – WPH Fed

Lane 7 – NEAA FAST

Lane 8 – NEAA Fed

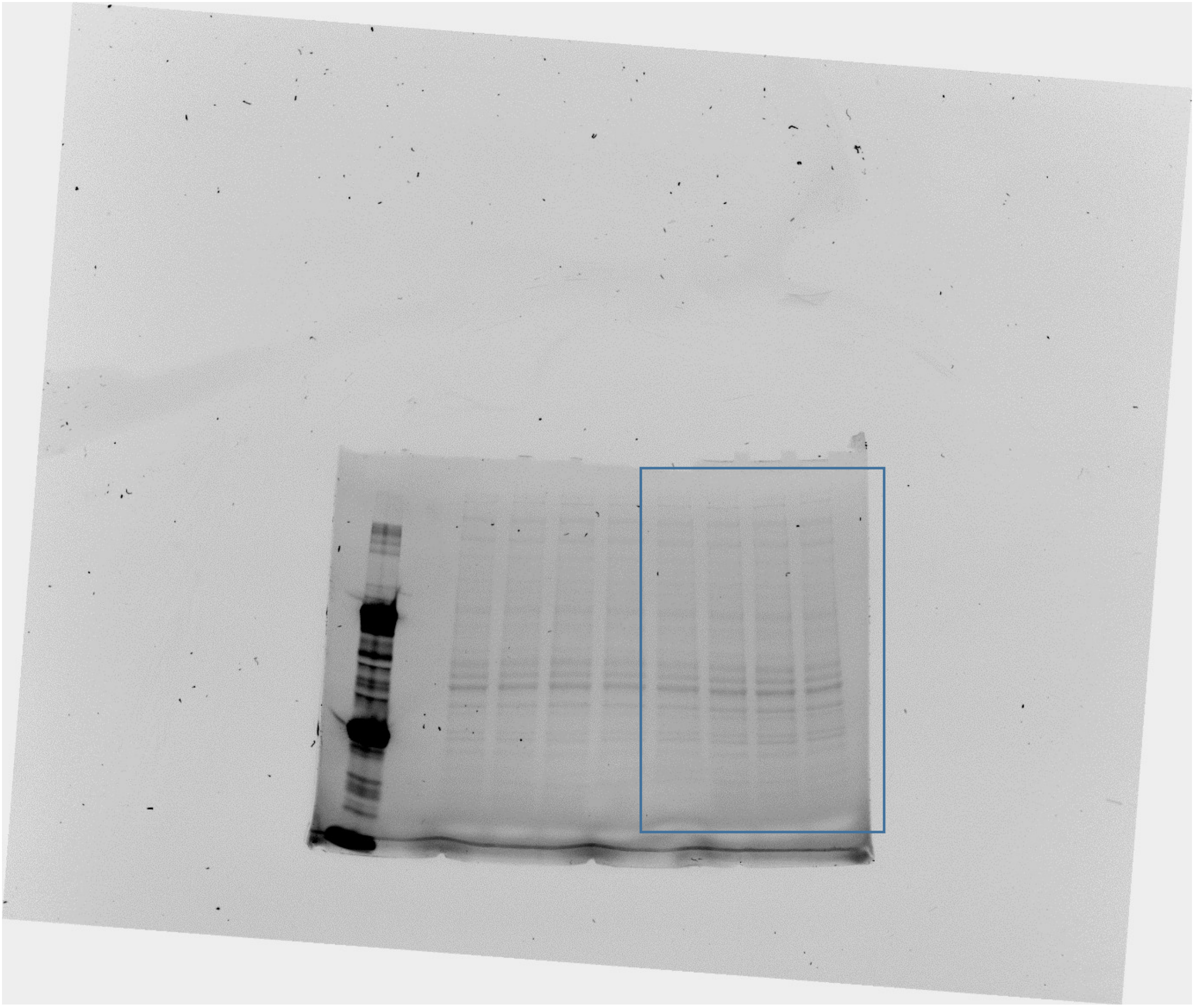

Total Protein Loading Control Participant 4 (Stain-free activated gel)

Lane 5 – WPH FAST

Lane 6 – WPH Fed

Lane 7 – NEAA FAST

Lane 8 – NEAA Fed

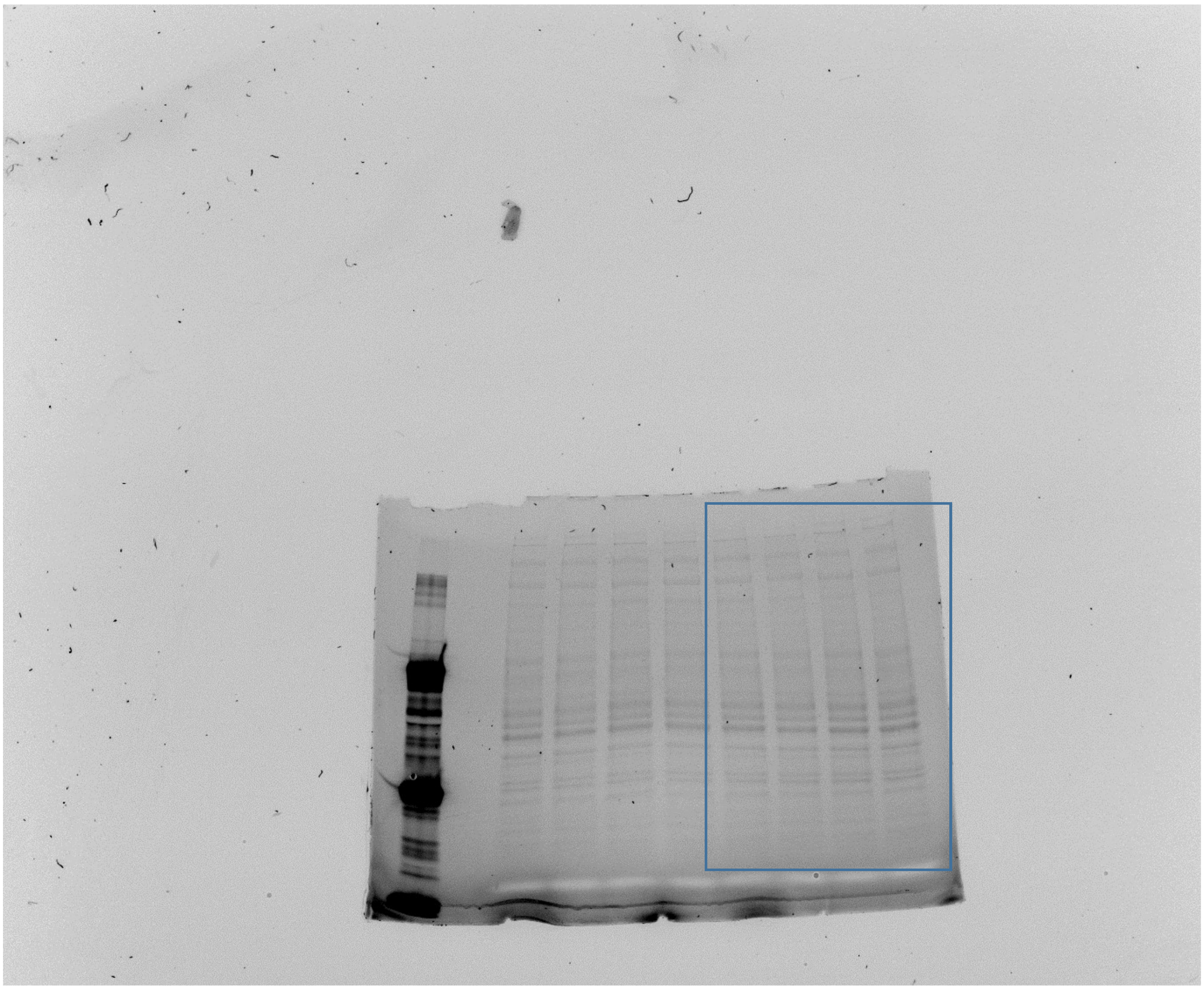

Total Protein Loading Control Participant 5 (Stain-free activated gel)

Lane 5 – WPH FAST

Lane 6 – WPH Fed

Lane 7 – NEAA FAST

Lane 8 – NEAA Fed

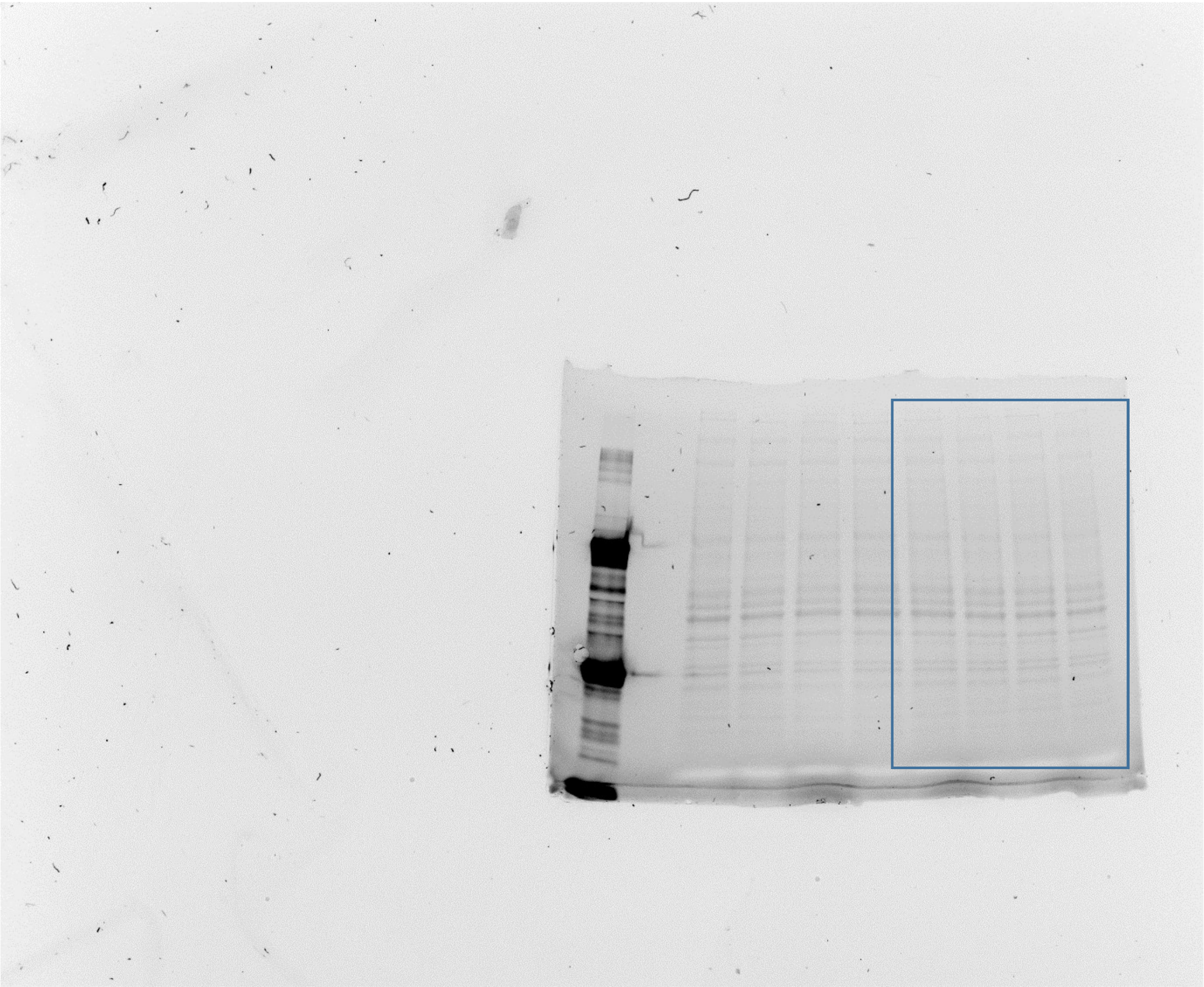

Total Protein Loading Control Participant 6 (Stain-free activated gel)

Lane 5 – WPH FAST

Lane 6 – WPH Fed

Lane 7 – NEAA FAST

Lane 8 – NEAA Fed

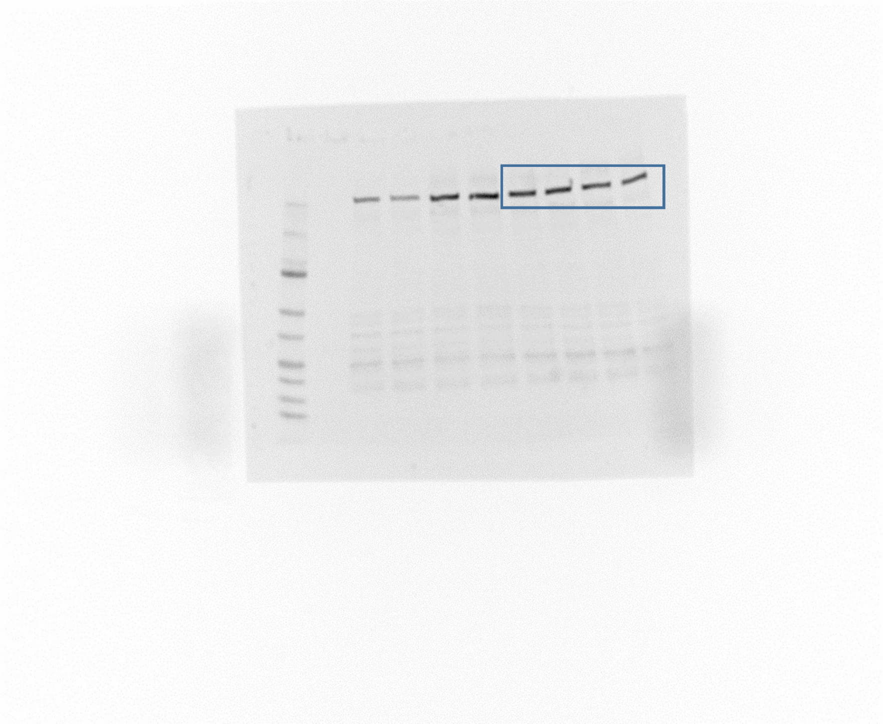

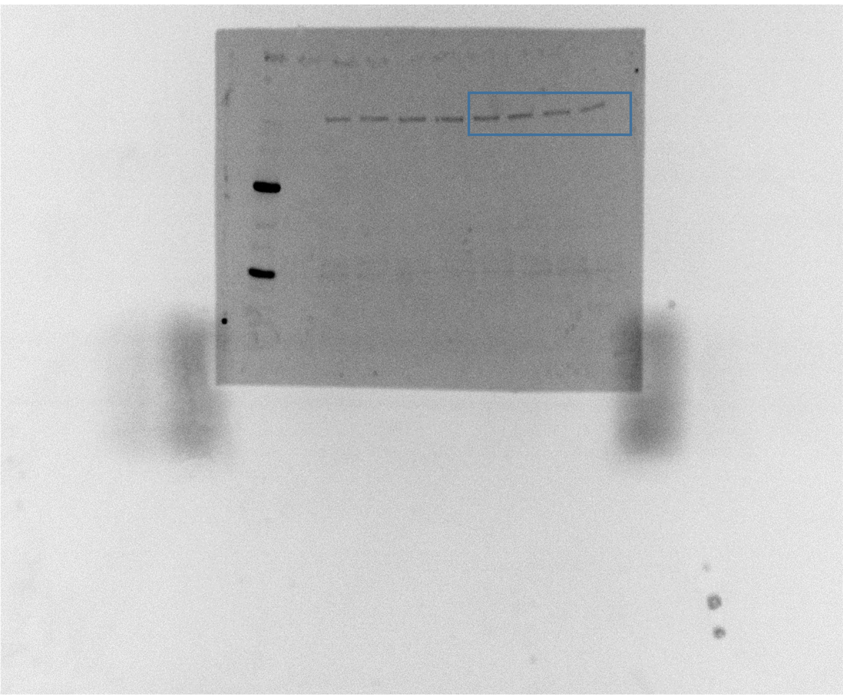

Phospho (above) and Total (below) mTOR Participant 1

Lane 5 – WPH FAST

Lane 6 – WPH Fed

Lane 7 – NEAA FAST

Lane 8 – NEAA Fed

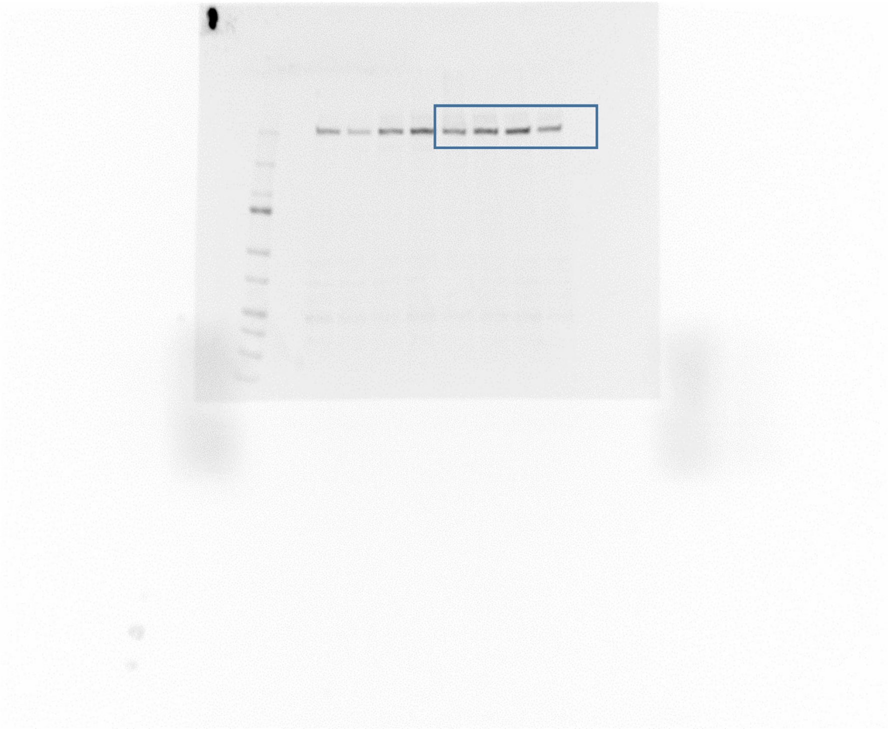

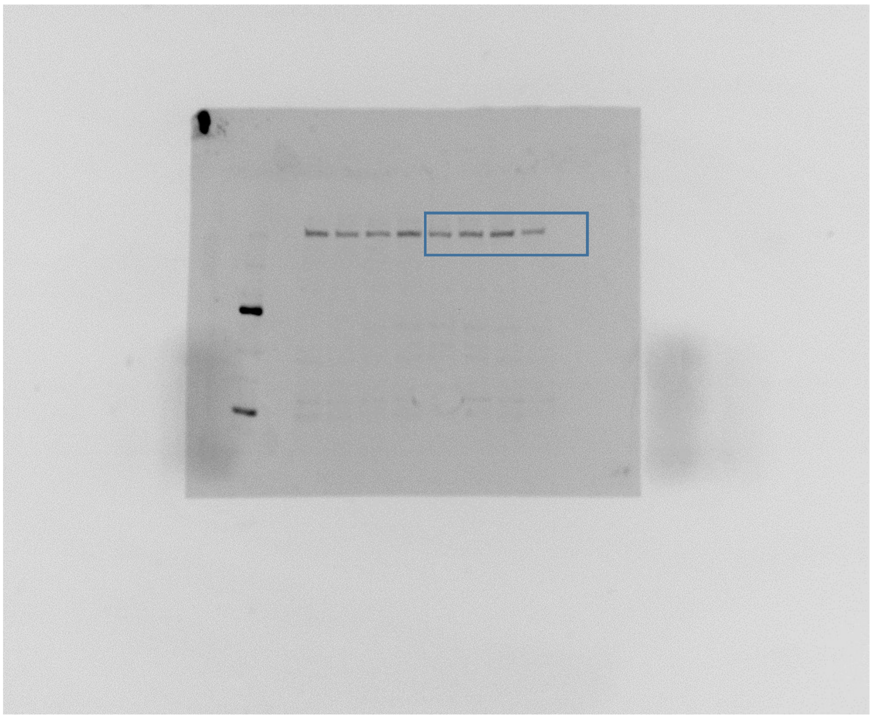

Phospho (above) and Total (below) mTOR Participant 2

Lane 5 – WPH FAST

Lane 6 – WPH Fed

Lane 7 – NEAA FAST

Lane 8 – NEAA Fed

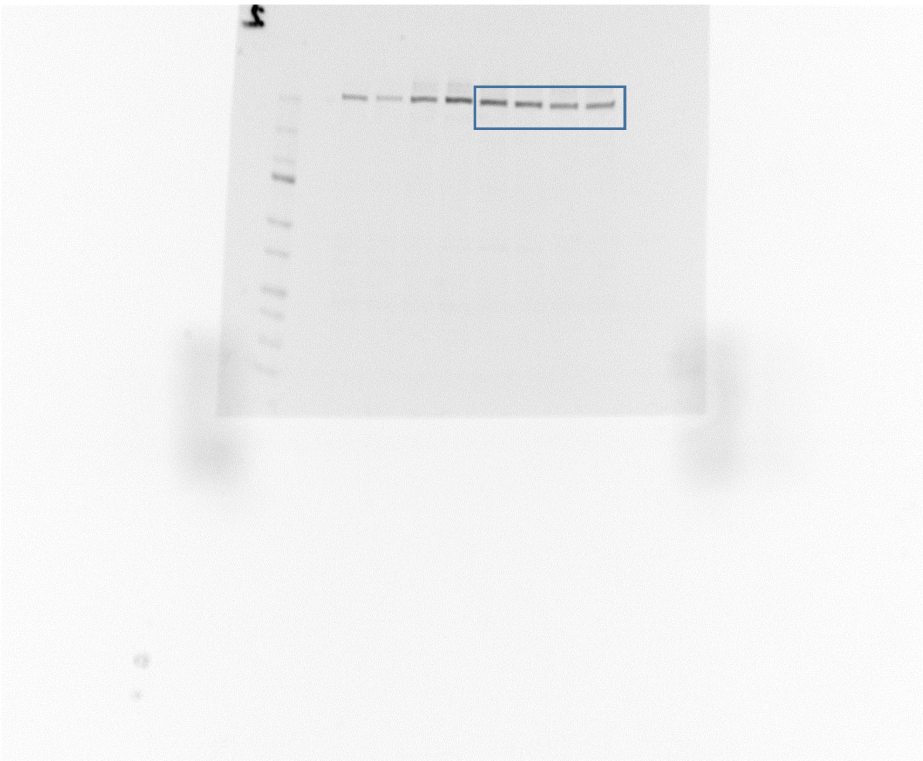

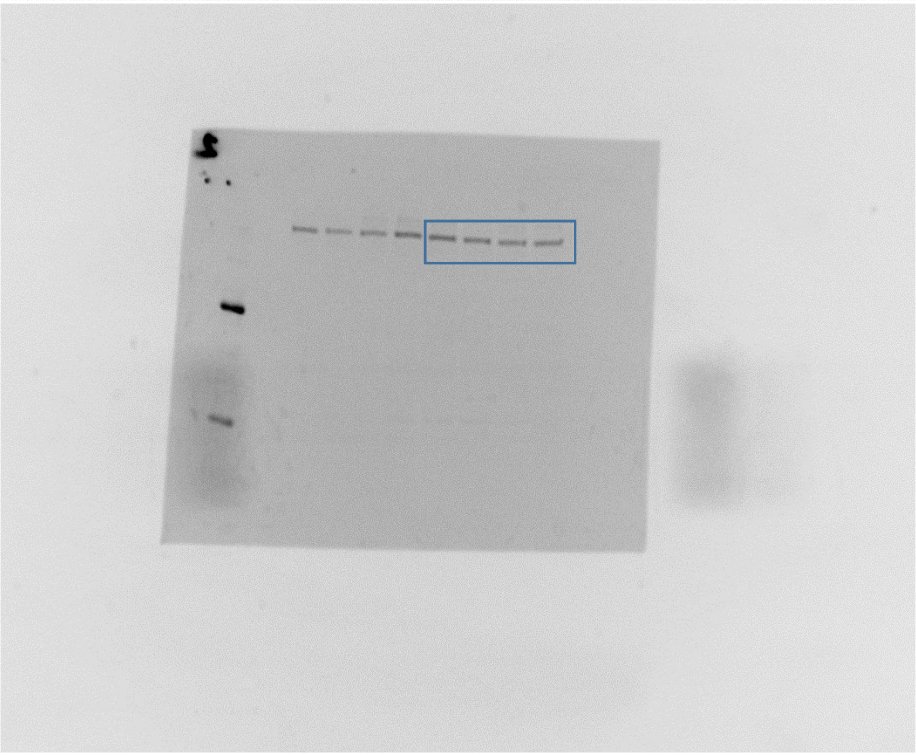

Phospho (above) and Total (below) mTOR Participant 3

Lane 5 – WPH FAST

Lane 6 – WPH Fed

Lane 7 – NEAA FAST

Lane 8 – NEAA Fed

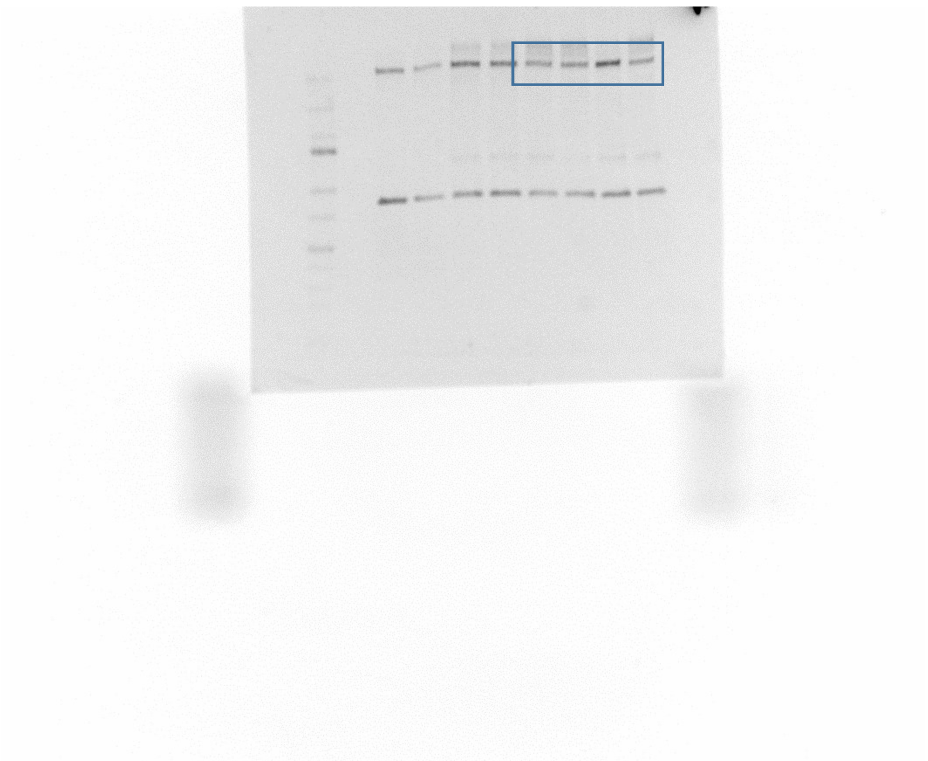

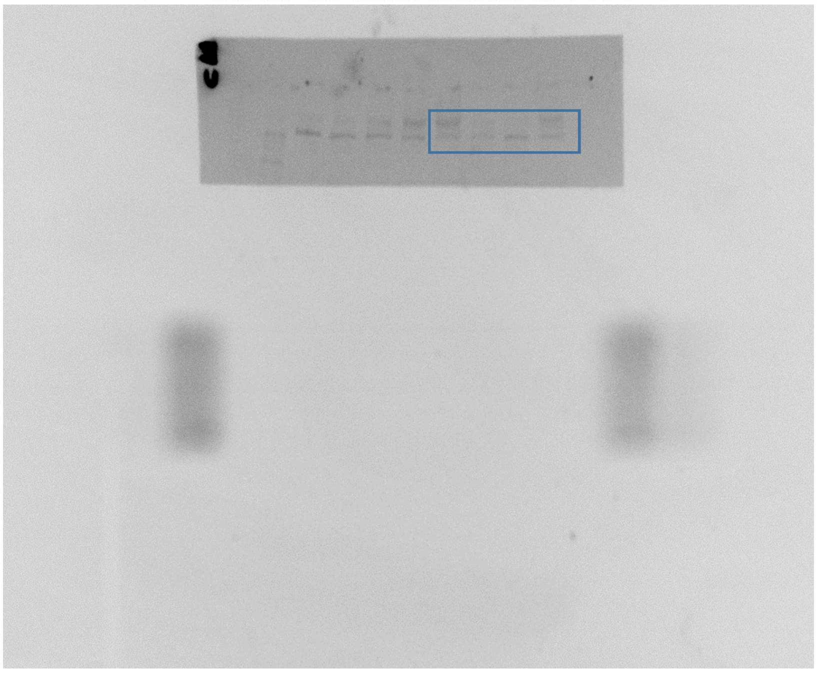

Phospho (above) and Total (below) mTOR Participant 4

Lane 5 – WPH FAST

Lane 6 – WPH Fed

Lane 7 – NEAA FAST

Lane 8 – NEAA Fed

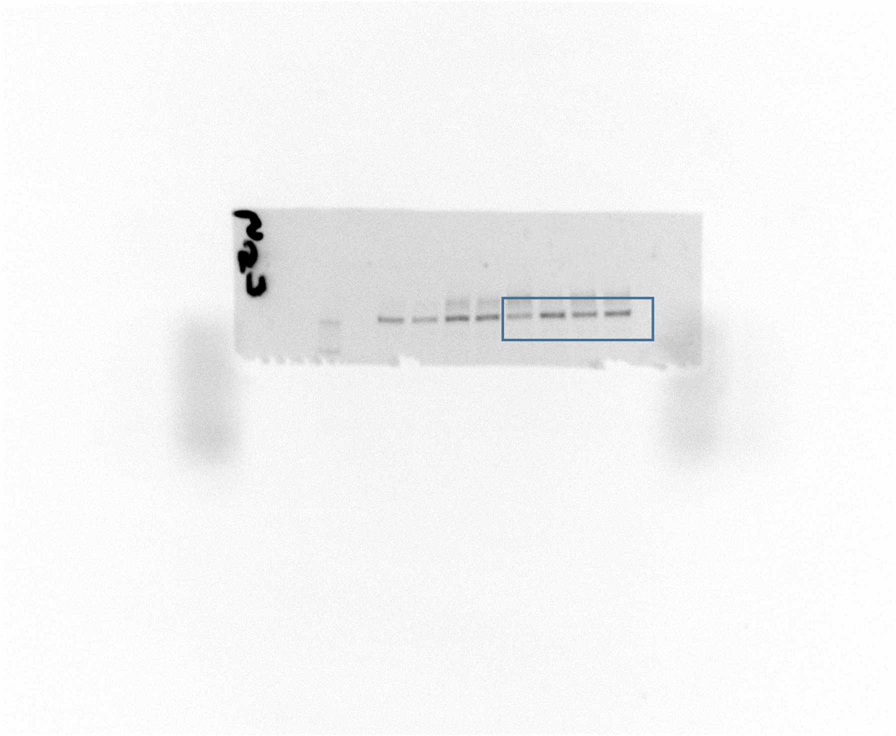

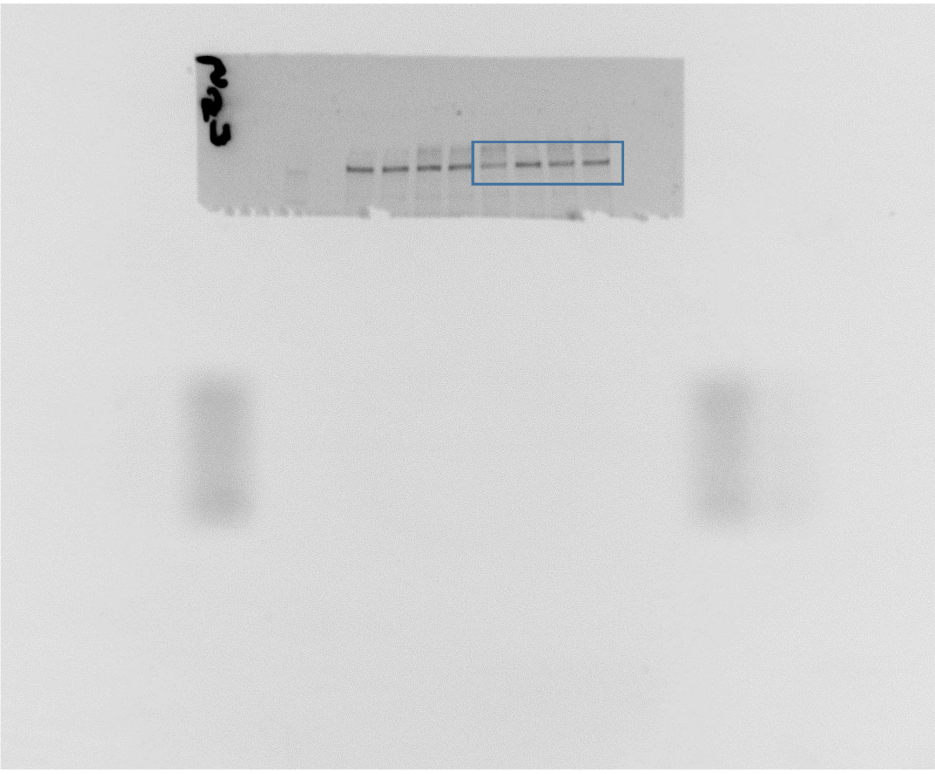

Phospho (above) and Total (below) mTOR Participant 5

Lane 5 – WPH FAST

Lane 6 – WPH Fed

Lane 7 – NEAA FAST

Lane 8 – NEAA Fed

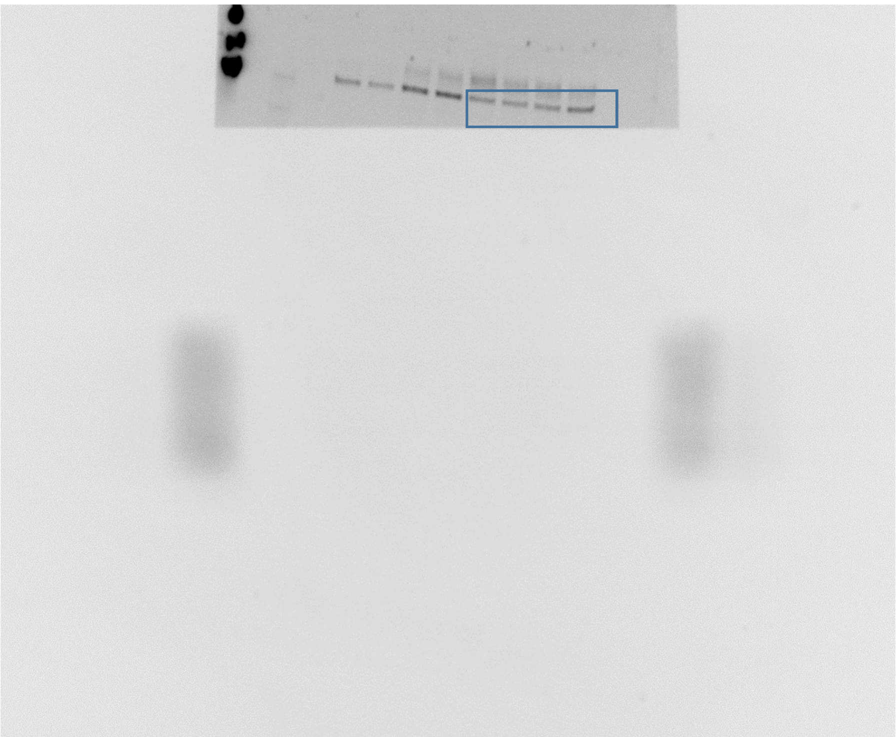

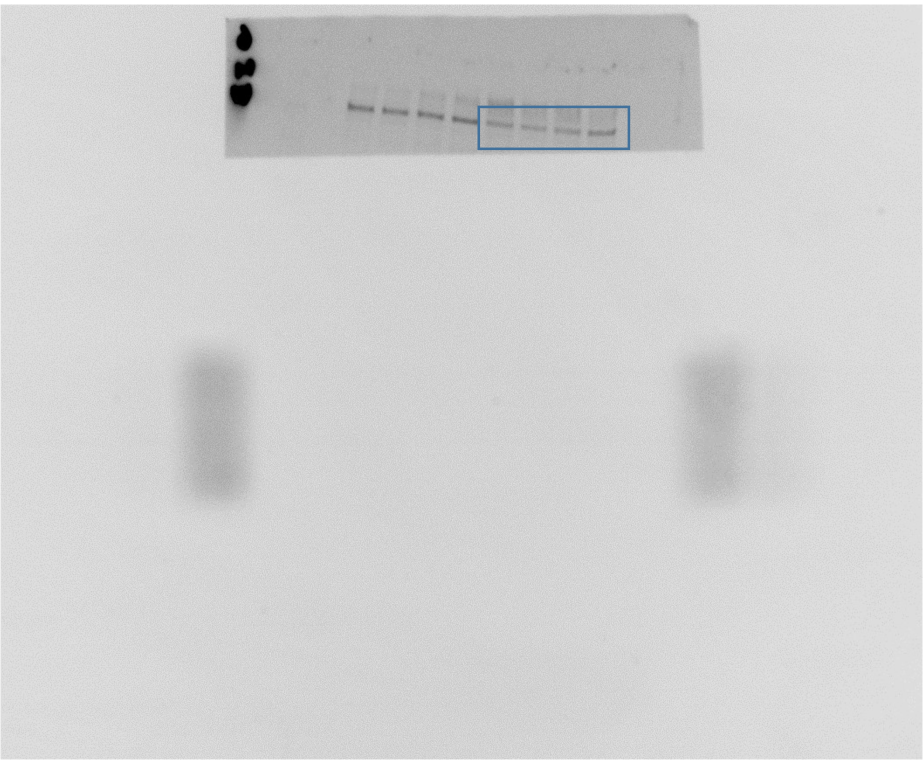

Phospho (above) and Total (below) mTOR Participant 6

Lane 5 – WPH FAST

Lane 6 – WPH Fed

Lane 7 – NEAA FAST

Lane 8 – NEAA Fed

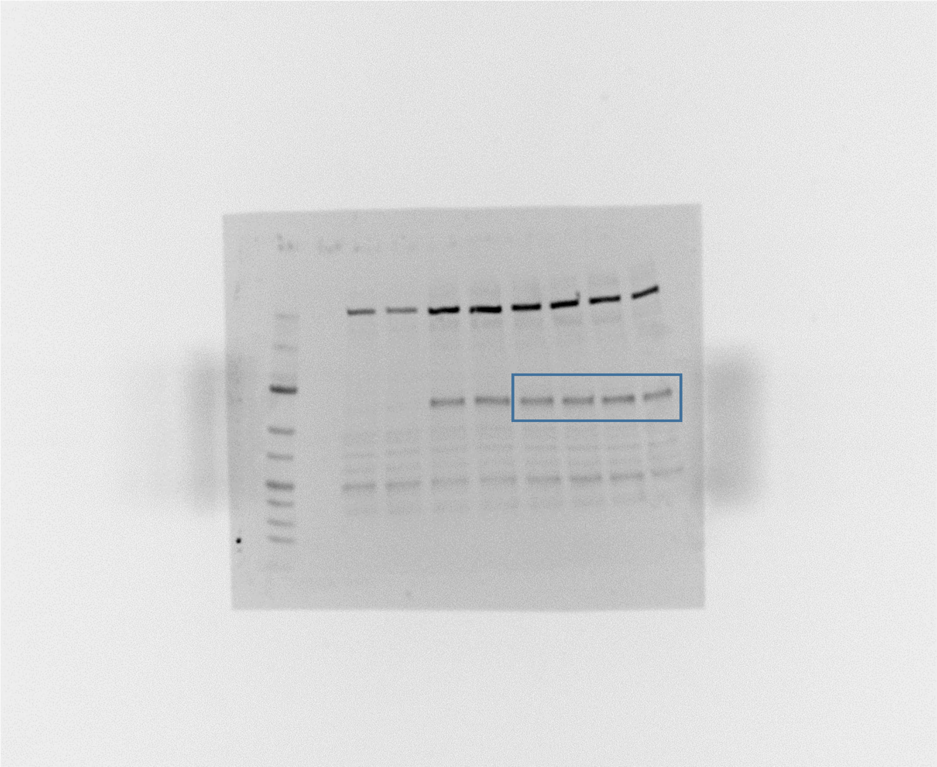

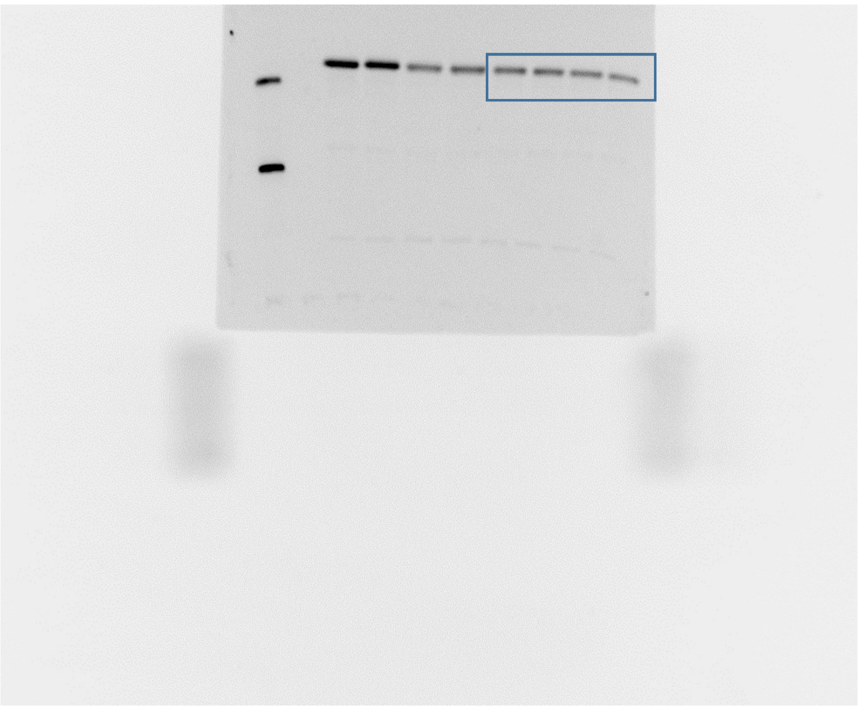

Phospho (above) and Total (below) P70S6K Participant 1

Lane 5 – WPH FAST

Lane 6 – WPH Fed

Lane 7 – NEAA FAST

Lane 8 – NEAA Fed

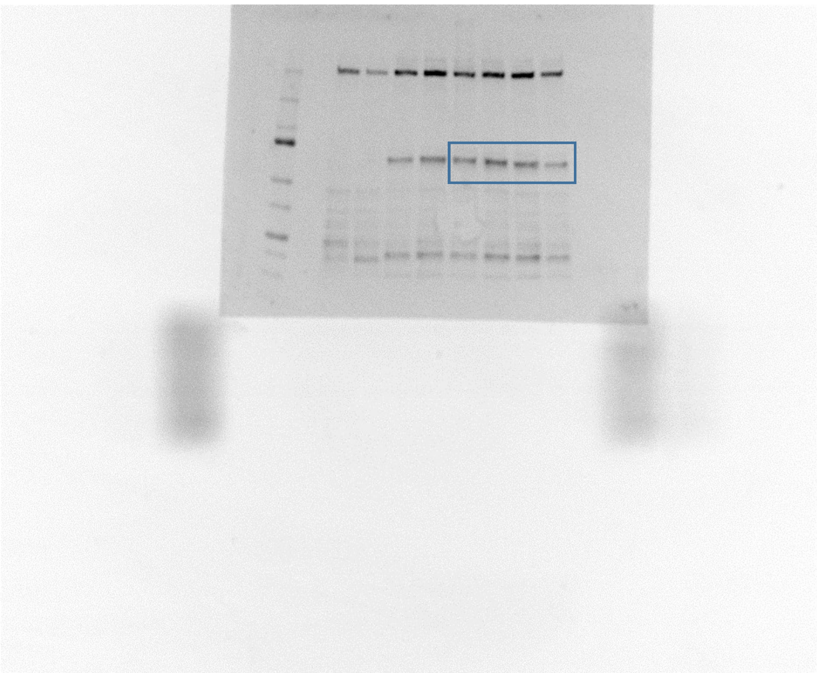

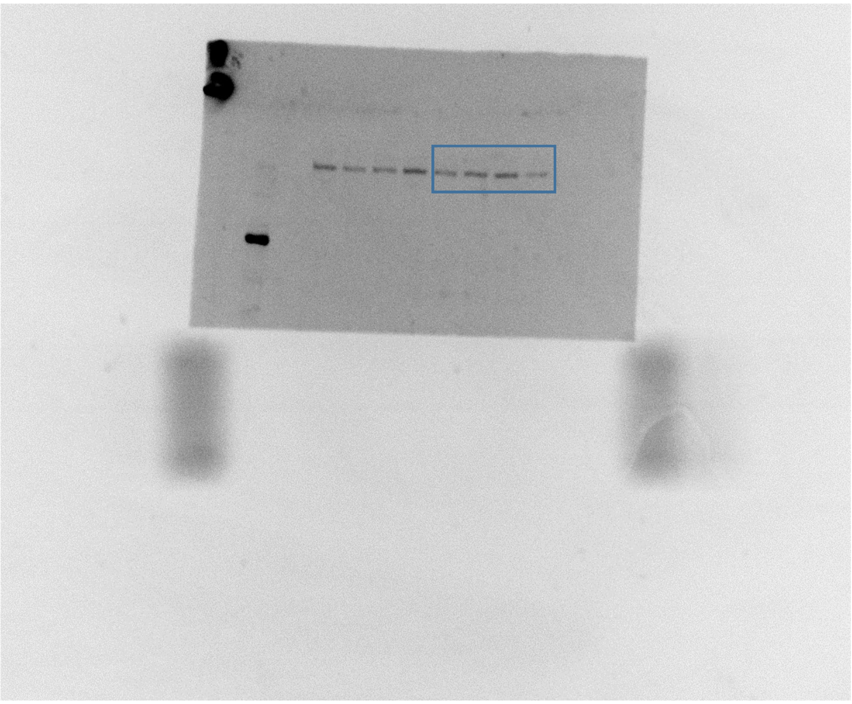

Phospho (above) and Total (below) P70S6K Participant 2

Lane 5 – WPH FAST

Lane 6 – WPH Fed

Lane 7 – NEAA FAST

Lane 8 – NEAA Fed

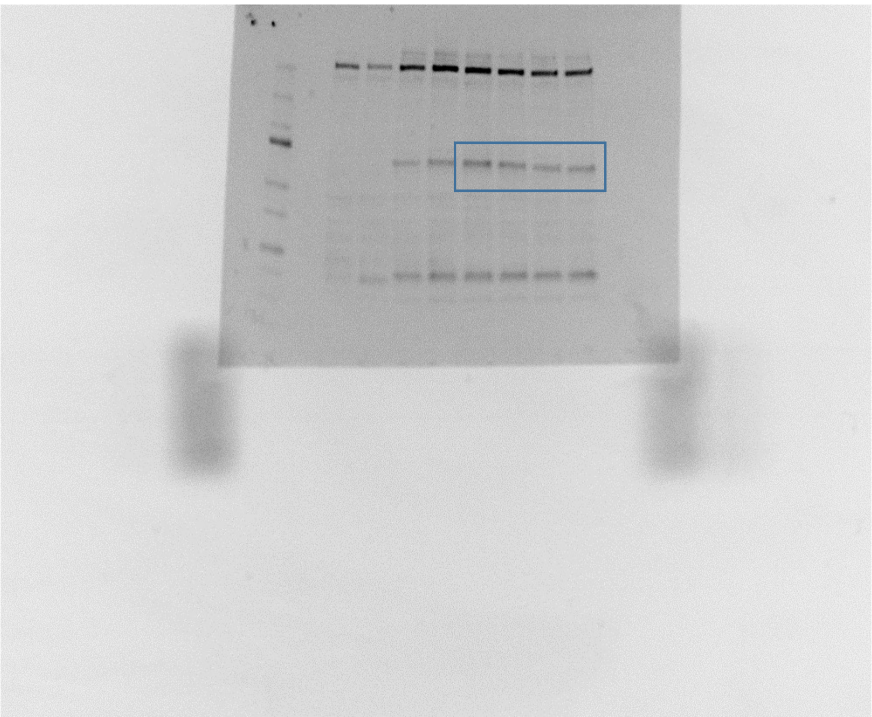

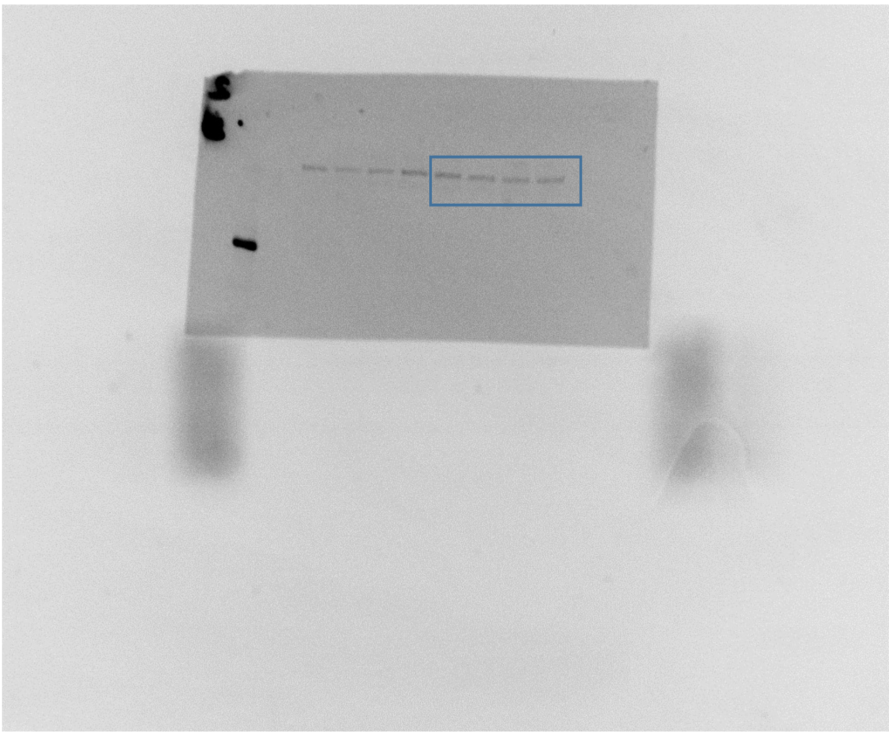

Phospho (above) and Total (below) P70S6K Participant 3

Lane 5 – WPH FAST

Lane 6 – WPH Fed

Lane 7 – NEAA FAST

Lane 8 – NEAA Fed

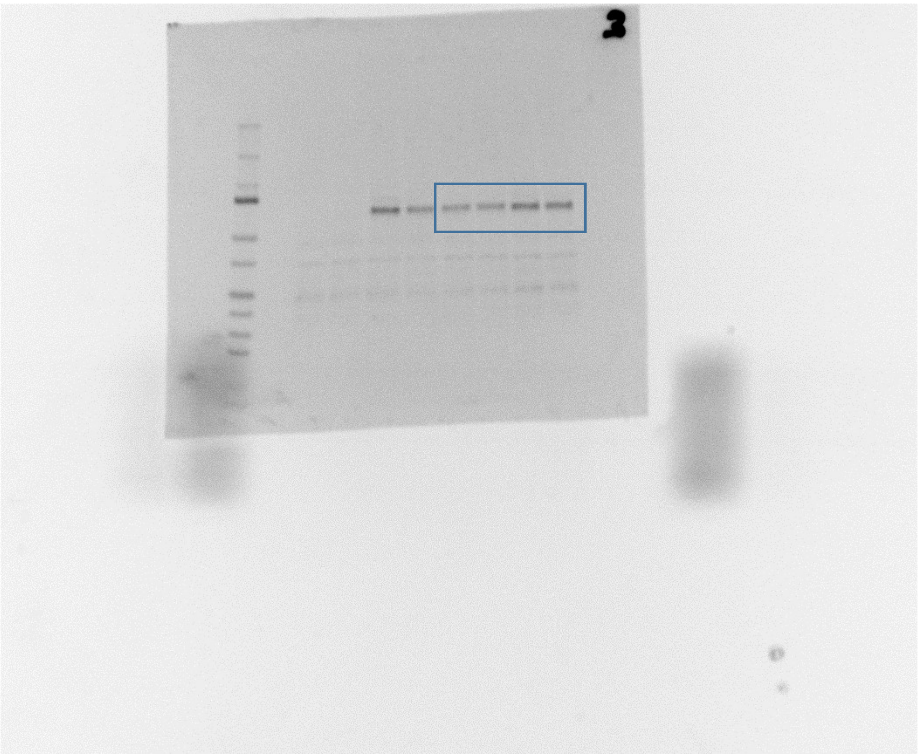

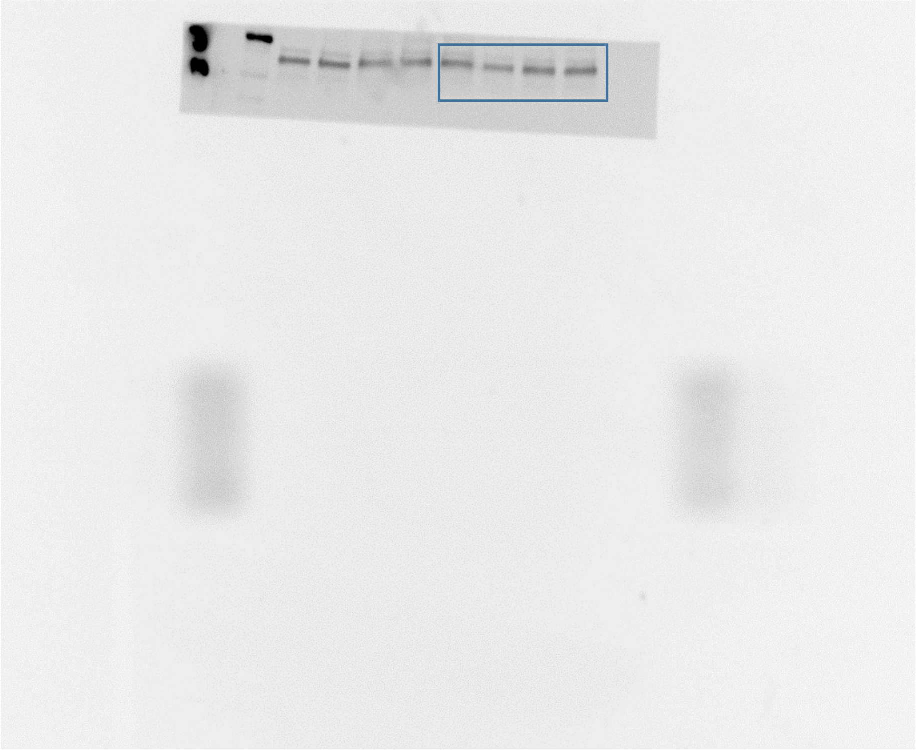

Phospho (above) and Total (below) P70S6K Participant 4

Lane 5 – WPH FAST

Lane 6 – WPH Fed

Lane 7 – NEAA FAST

Lane 8 – NEAA Fed

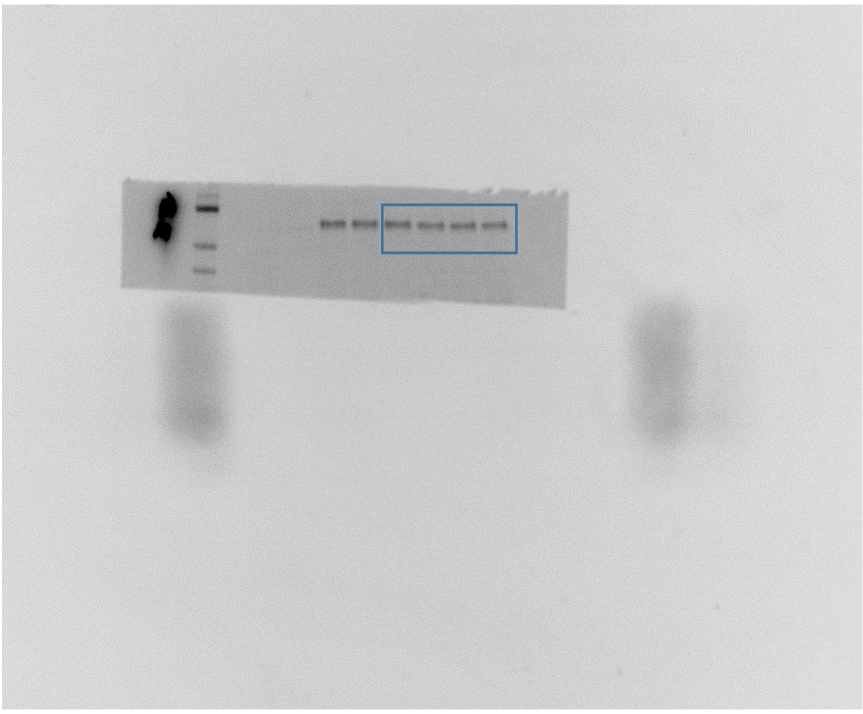

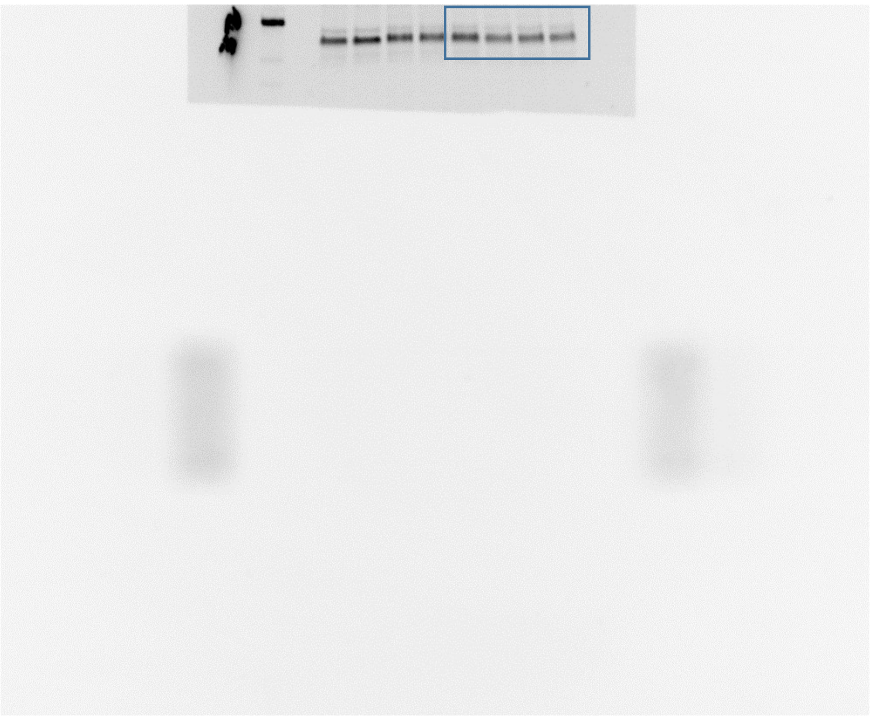

Phospho (above) and Total (below) P70S6K Participant 5

Lane 5 – WPH FAST

Lane 6 – WPH Fed

Lane 7 – NEAA FAST

Lane 8 – NEAA Fed

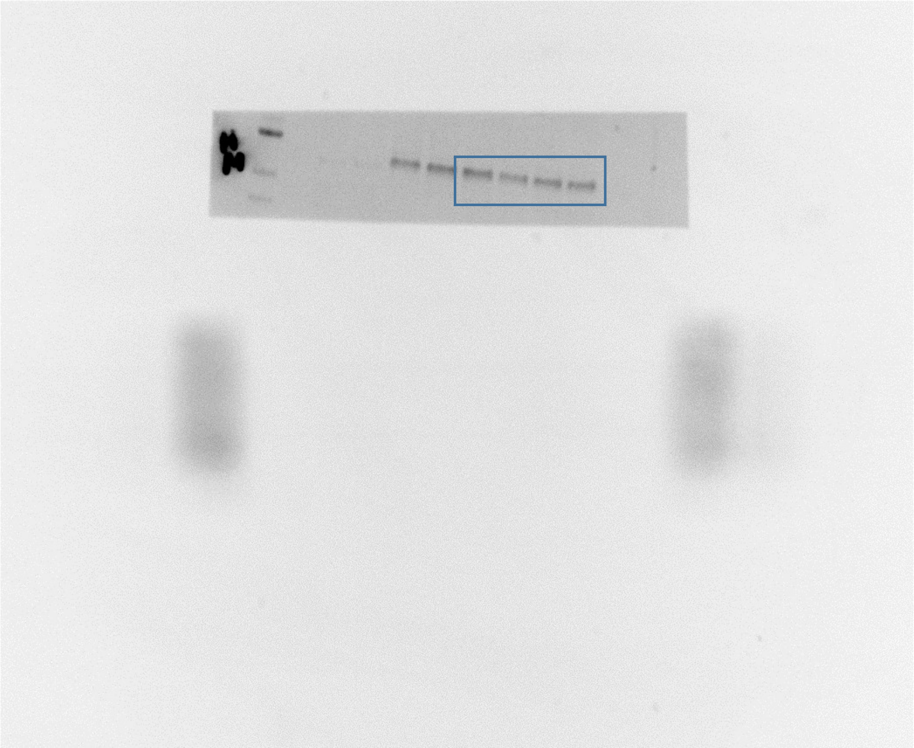

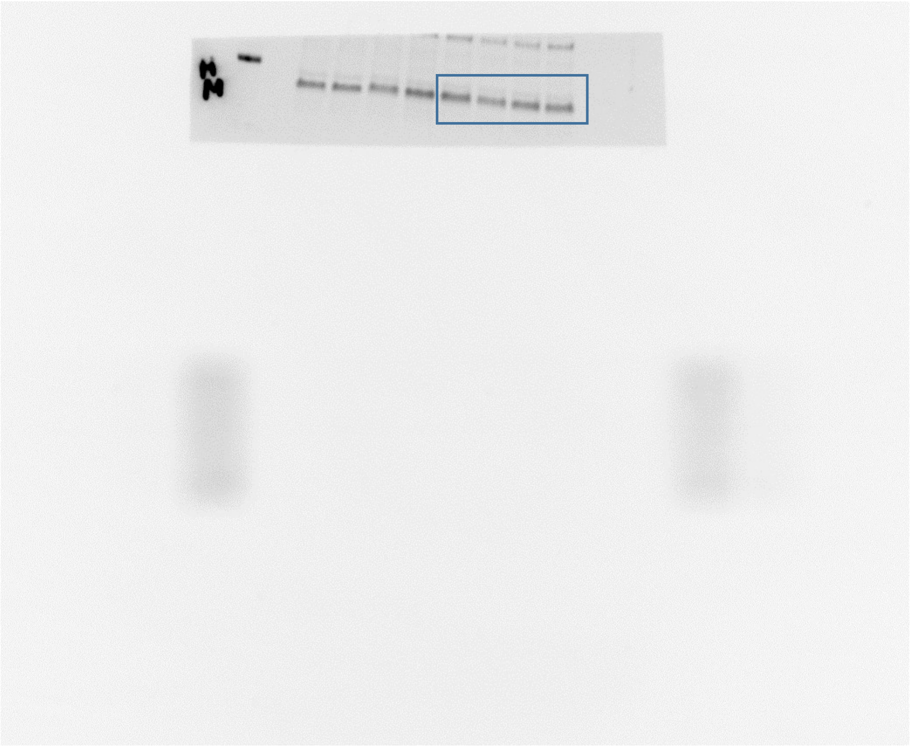

Phospho (above) and Total (below) P70S6K Participant 6

Lane 5 – WPH FAST

Lane 6 – WPH Fed

Lane 7 – NEAA FAST

Lane 8 – NEAA Fed

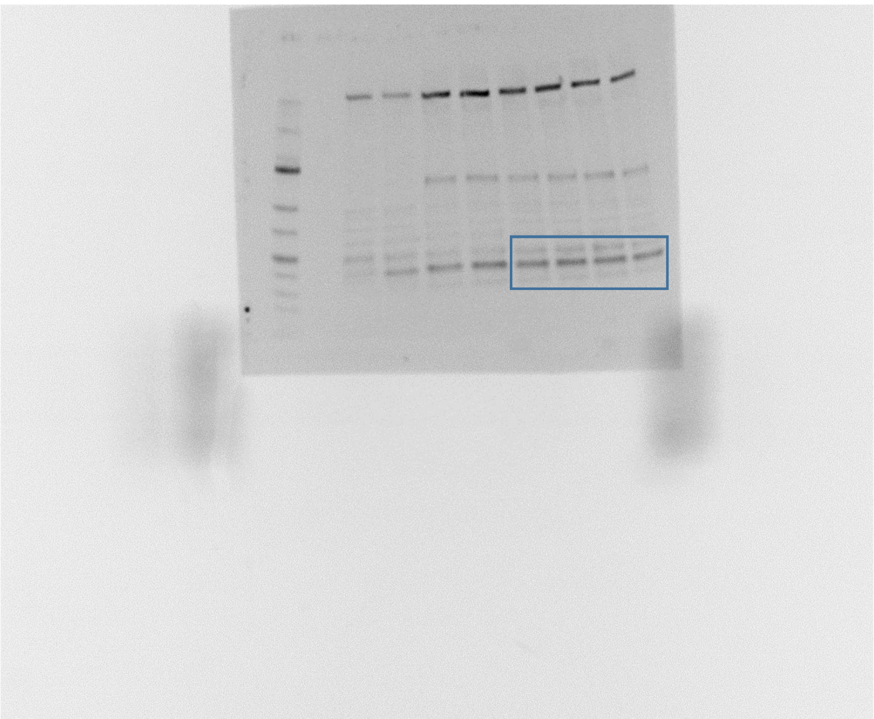

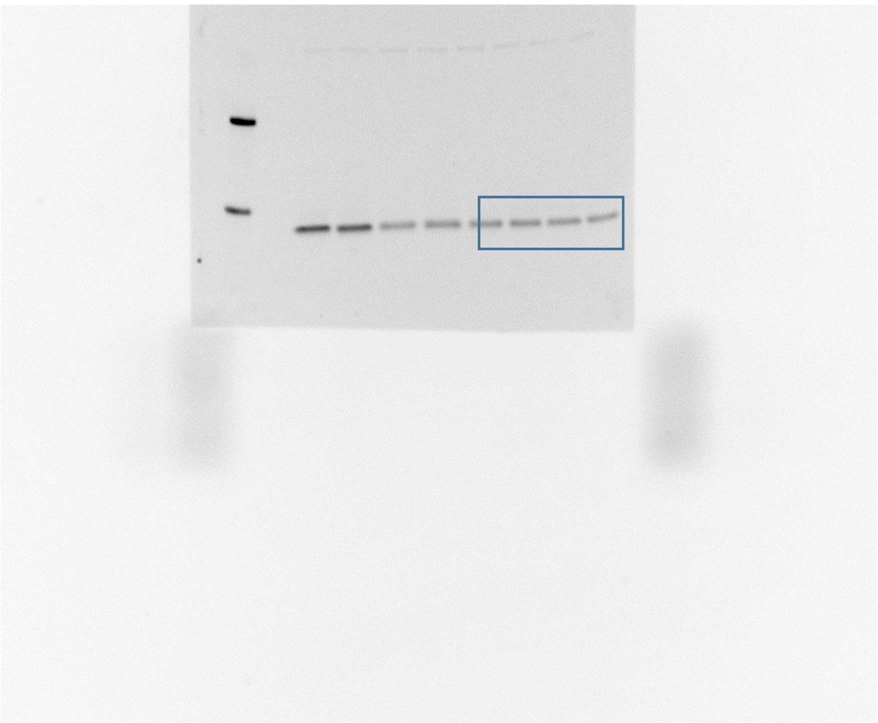

Phospho (above) and Total (below) 4E-BP1 Participant 1

Lane 5 – WPH FAST

Lane 6 – WPH Fed

Lane 7 – NEAA FAST

Lane 8 – NEAA Fed

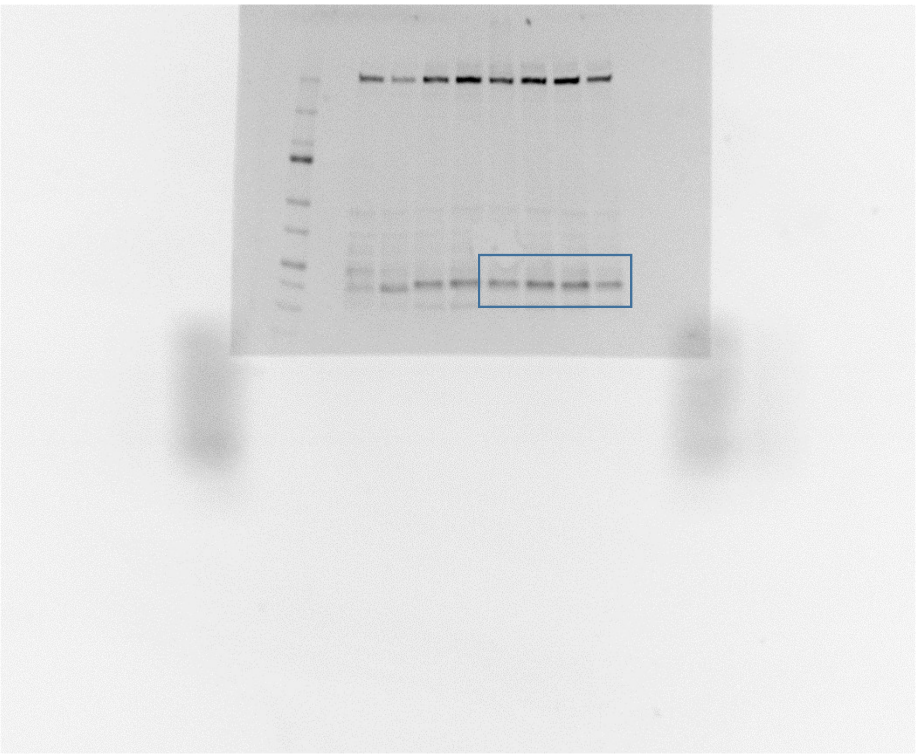

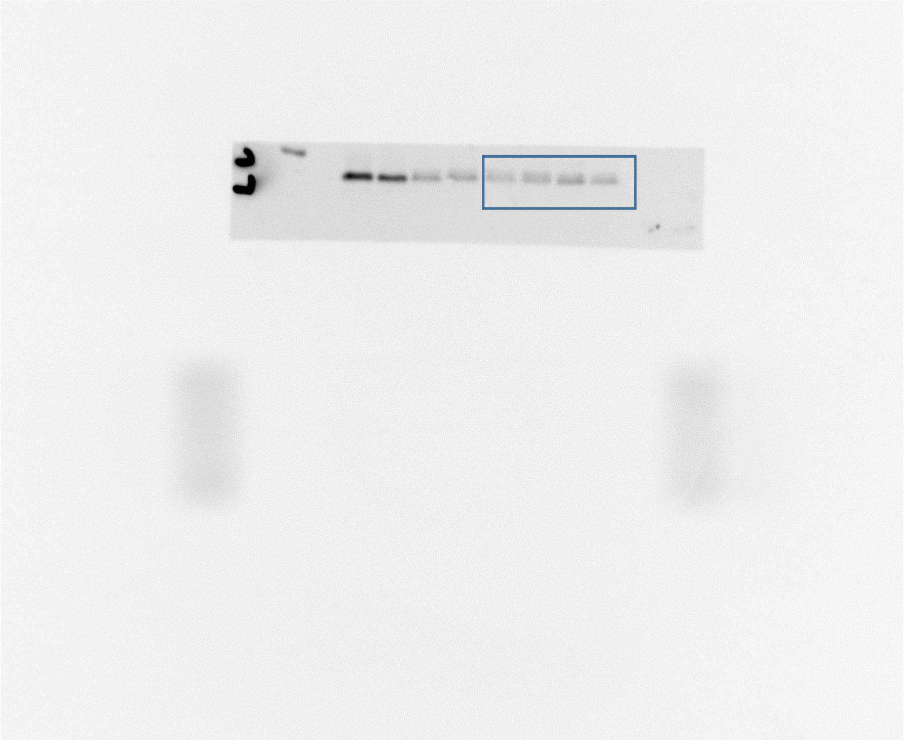

Phospho (above) and Total (below) 4E-BP1 Participant 2

Lane 5 – WPH FAST

Lane 6 – WPH Fed

Lane 7 – NEAA FAST

Lane 8 – NEAA Fed

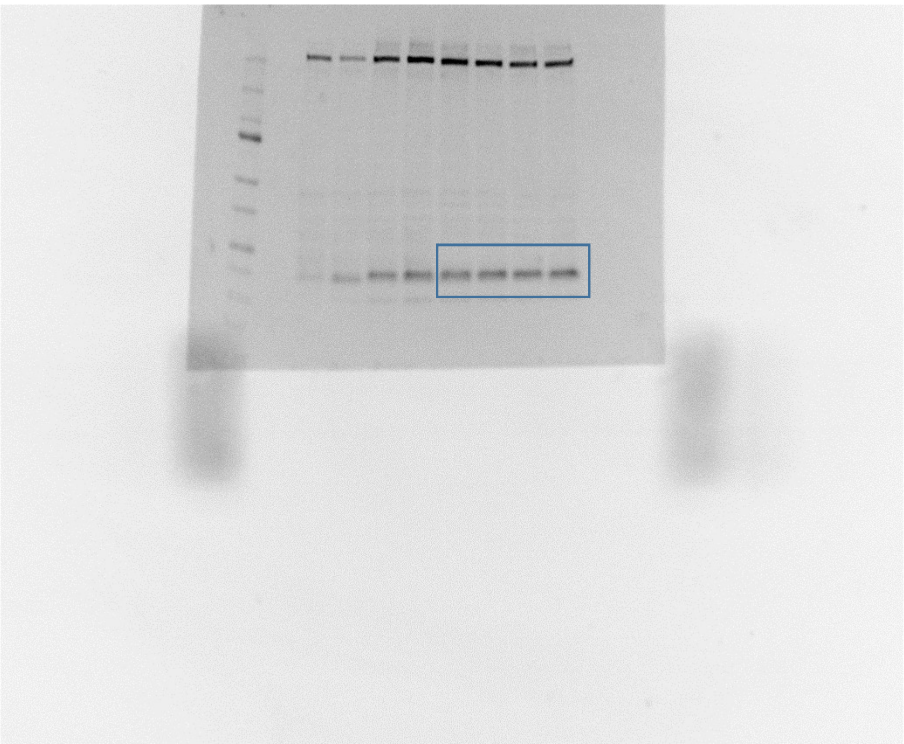

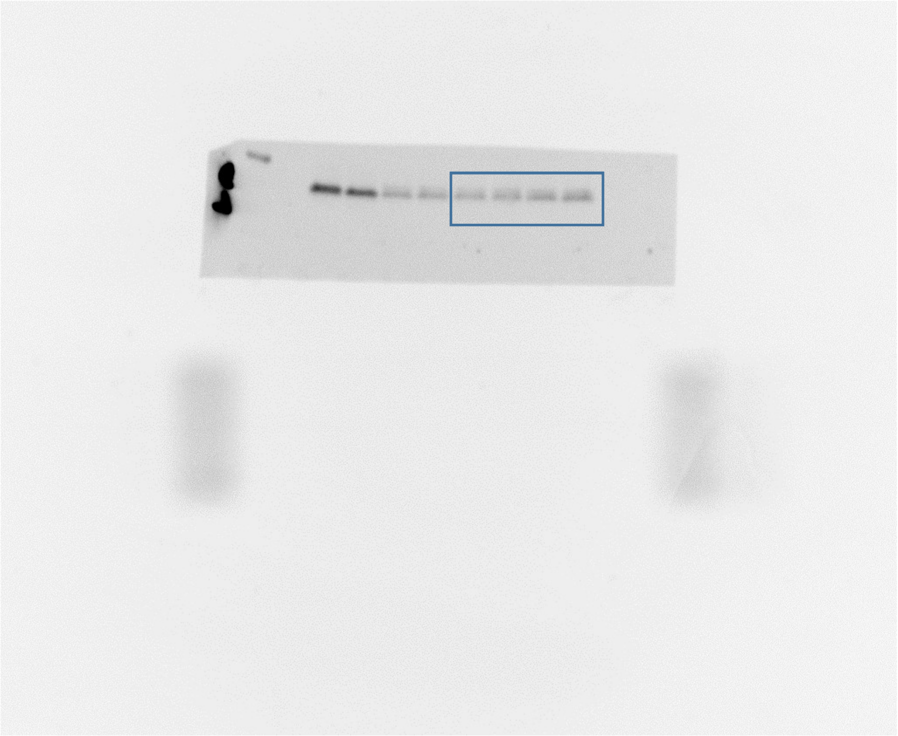

Phospho (above) and Total (below) 4E-BP1 Participant 3

Lane 5 – WPH FAST

Lane 6 – WPH Fed

Lane 7 – NEAA FAST

Lane 8 – NEAA Fed

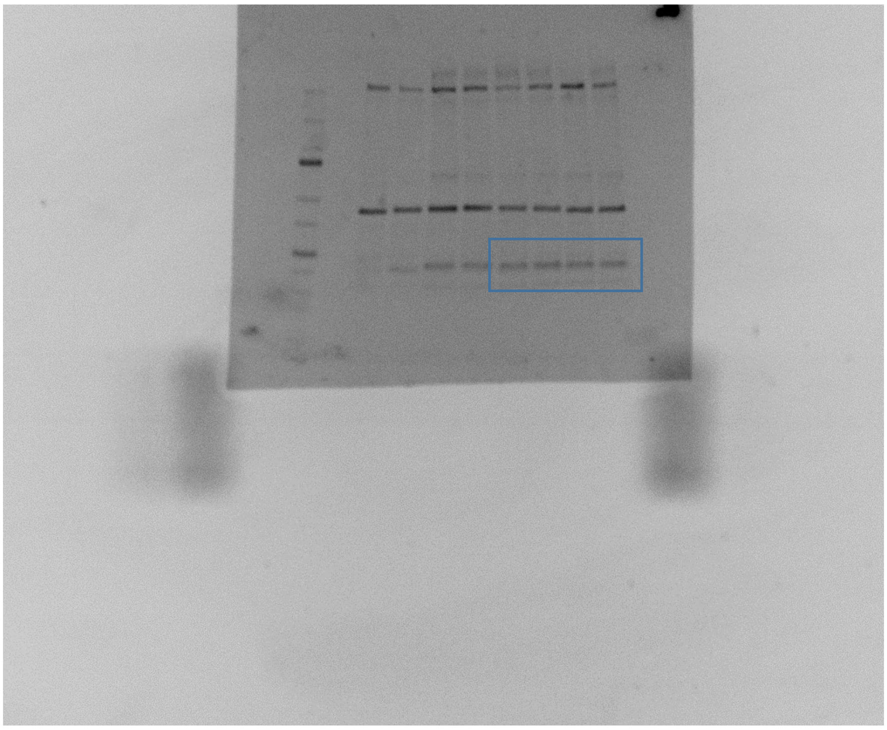

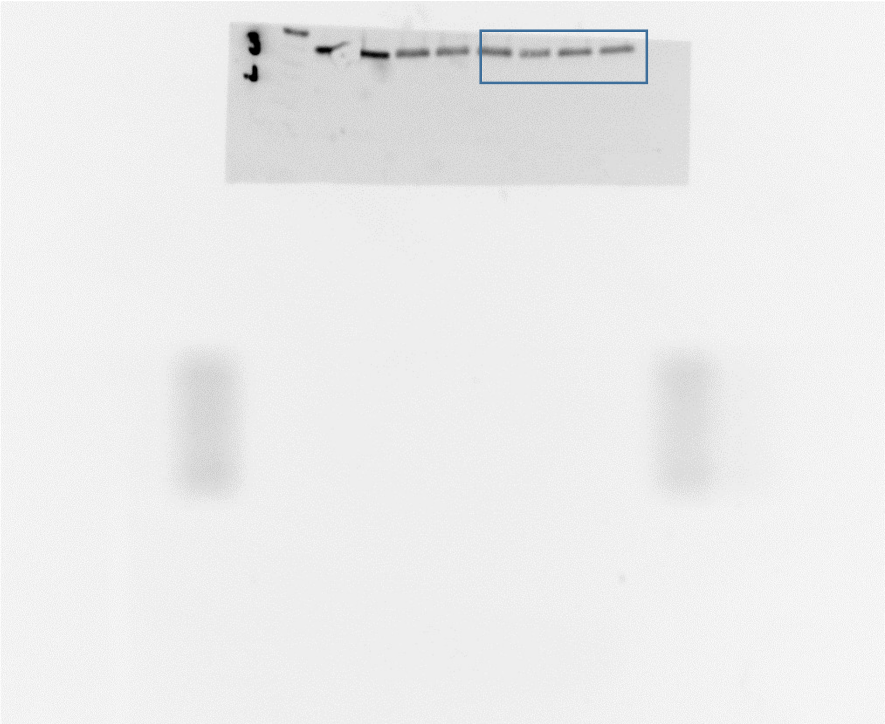

Phospho (above) and Total (below) 4E-BP1 Participant 4

Lane 5 – WPH FAST

Lane 6 – WPH Fed

Lane 7 – NEAA FAST

Lane 8 – NEAA Fed

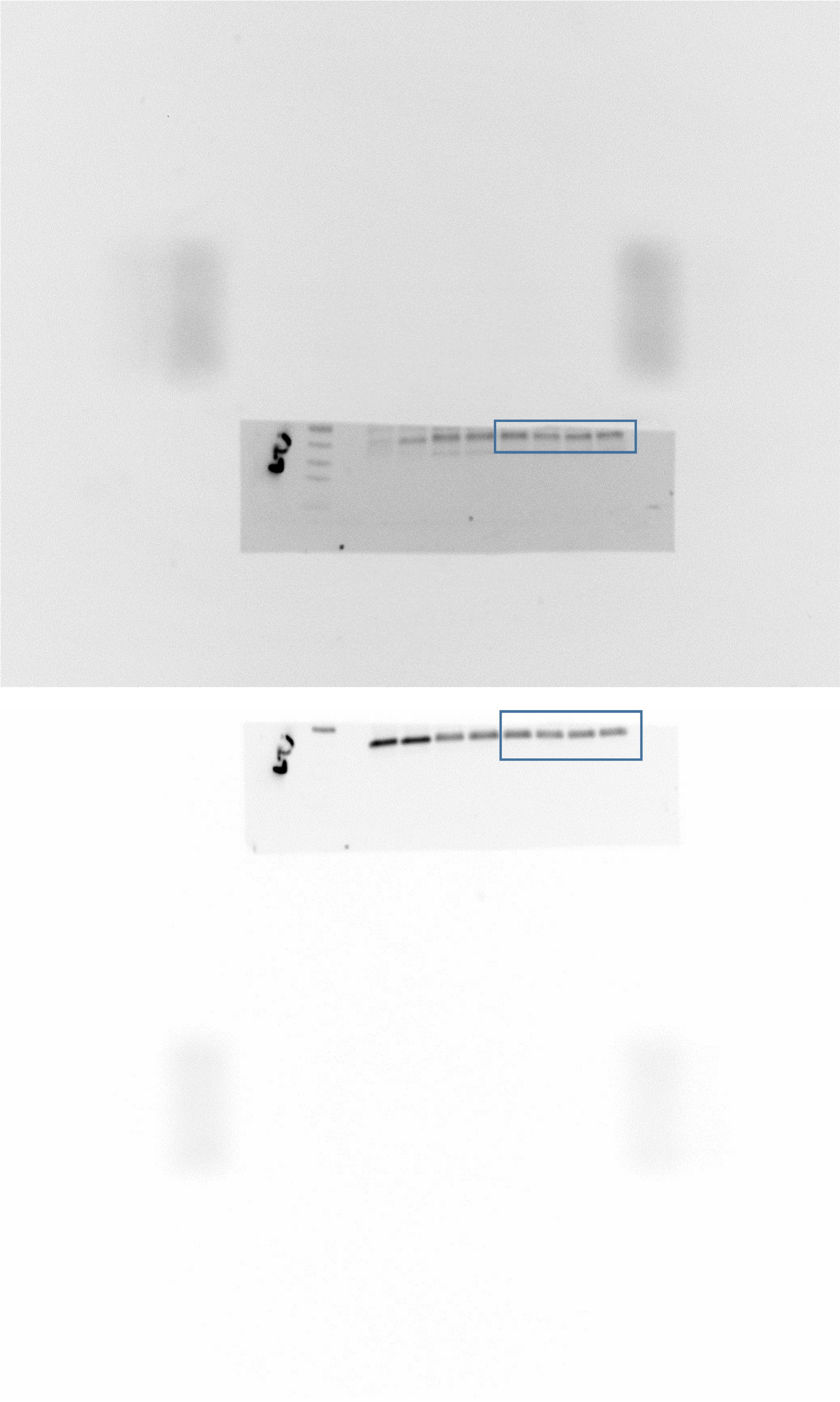

Phospho (above) and Total (below) 4E-BP1 Participant 5

Lane 5 – WPH FAST

Lane 6 – WPH Fed

Lane 7 – NEAA FAST

Lane 8 – NEAA Fed

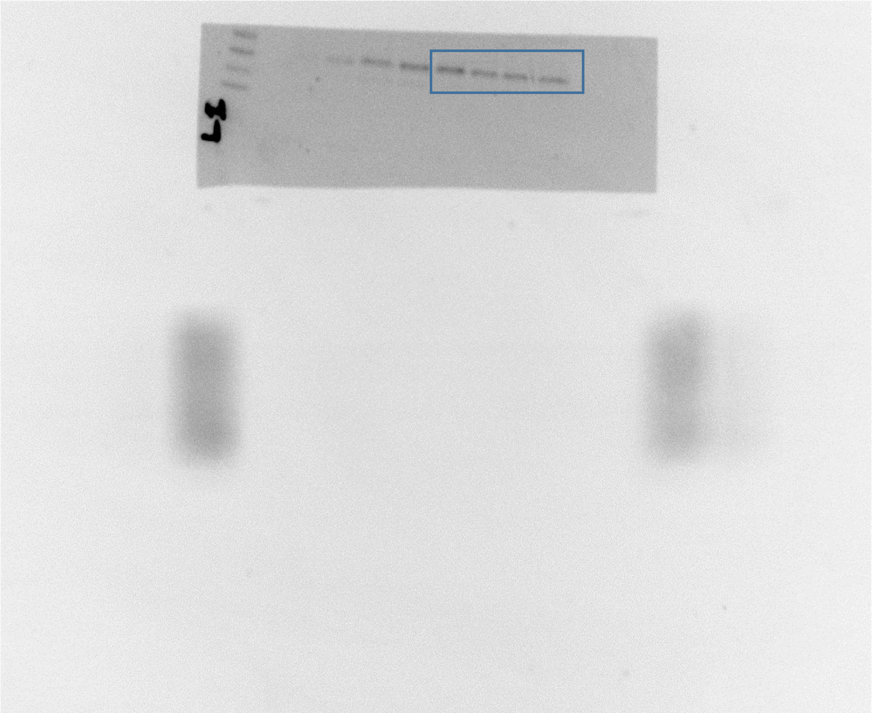

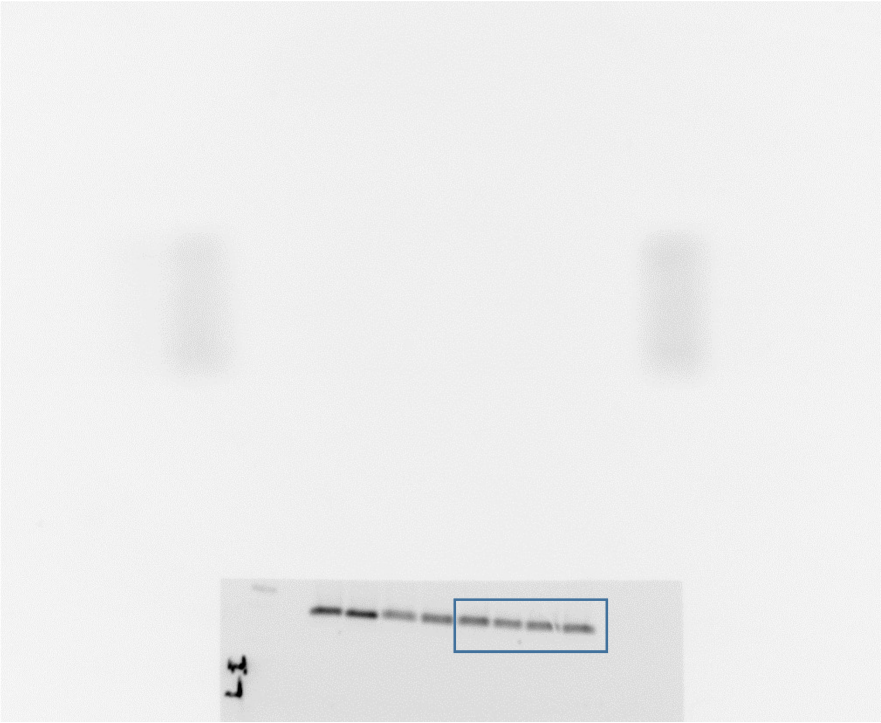

Phospho (above) and Total (below) 4E-BP1 Participant 6

Lane 5 – WPH FAST

Lane 6 – WPH Fed

Lane 7 – NEAA FAST

Lane 8 – NEAA Fed
